# Calcium activation mechanism of a noncanonical aromatic L-amino acid decarboxylase from psilocybin mushroom *Psilocybe cubensis*

**DOI:** 10.1038/s42003-026-09756-y

**Published:** 2026-02-26

**Authors:** Tianjie Li, Erin E. Reynolds, Ziqi Wang, Michael P. Torrens-Spence, Jing-Ke Weng, Yi Wang

**Affiliations:** 1 Department of Physics, The Chinese University of Hong Kong, Hong Kong SAR, China; 2 Institute for Plant-Human Interface, Northeastern University, Boston, MA USA; 3 Department of Chemical Engineering, Massachusetts Institute of Technology, Cambridge, MA USA; 4 Whitehead Institute for Biomedical Research, Cambridge, MA USA; 5 Department of Chemistry and Chemical Biology, Department of Bioengineering, and Department of Chemical Engineering, Northeastern University, Boston, MA USA

**Keywords:** Computational biophysics, Computational chemistry

## Abstract

*Pc*ncAAAD is a noncanonical fungal aromatic L-amino acid decarboxylase (AAAD) featuring a unique appendage C-terminal domain (CTD) and two metal-binding sites. Unlike its mammalian and plant counterparts, *Pc*ncAAAD is activated by calcium, although the exact activation mechanism remains unclear. Here, we establish an in silico RMSD-based evaluation model through molecular dynamics simulations, validated by in vitro enzyme assays, to decipher the enzyme’s calcium activation mechanism. The metal-binding site at the intra-monomer interface between the N-terminal domain and the CTD (site A) is found to play a primary role in the calcium activation of *Pc*ncAAAD, whereas the secondary site within the unique CTD (site B) contributes to the calcium-mediated stabilization of enzyme structure. Binding of calcium, but not sodium, exerts a profound influence on *Pc*ncAAAD activity by stabilizing a “lid-rim” structure underlying site A, which in turn maintains the integrity of the substrate-binding environment. In silico mutations disrupting site A or the lid-rim structure show severe structural distortion of the active site, leading to reduced or even eliminated activity as demonstrated by in vitro assays. These findings deepen our understanding of metal-activatable enzymes and hold promise for the rational design of engineered enzymes for the synthesis of aromatic amino acid derivatives.

## Abstract (web summary)

Molecular dynamics simulations, an in silico scoring scheme and in vitro enzyme assays decipher the calcium activation mechanism of a noncanonical fungal aromatic L-amino acid decarboxylase.

## Introduction

Aromatic L-amino acid decarboxylases (AAADs) are a class of ancient pyridoxal 5’-phosphate (PLP)-dependent enzymes found in all domains of life involved in anabolic pathways for the production of various bioactive arylalkylamine and arylalkyladehyde derivatives^1–3^. Despite their taxonomic breadth, AAADs share extensive sequence identity as well as substantial structural and kinetic similarities^4,5^. Unlike other identified mammalian and plant AAADs, the noncanonical AAAD from *Psilocybe cubensis* (*Pc*ncAAAD) can be activated by calcium and contains a novel appendage C-terminal domain (CTD)^6^. *Pc*ncAAAD displays a broad substrate selectivity towards both proteinogenic and non-proteinogenic aromatic amino acids, including L-phenylalanine, L-tyrosine, L-tryptophan and L-kynurenine, and is capable of catalyzing both decarboxylation and latent decarboxylation-dependent oxidative deamination activities towards L-kynurenine and 3-hydroxy-L-kynurenine^6,7^. While its exact function in hallucinogenic mushroom is yet unknown, this enzyme exhibits catalytic activity analogous to the *Psilocybe cubensis* L-tryptophan decarboxylase (PsiD), which catalyzes the conversion of L-tryptophan to L-tryptamine in the biosynthesis of psilocybin, a tryptamine-derived psychedelic prodrug with potential application as antidepressants and anxiolytics^8–10^.

**Fig. 1:**
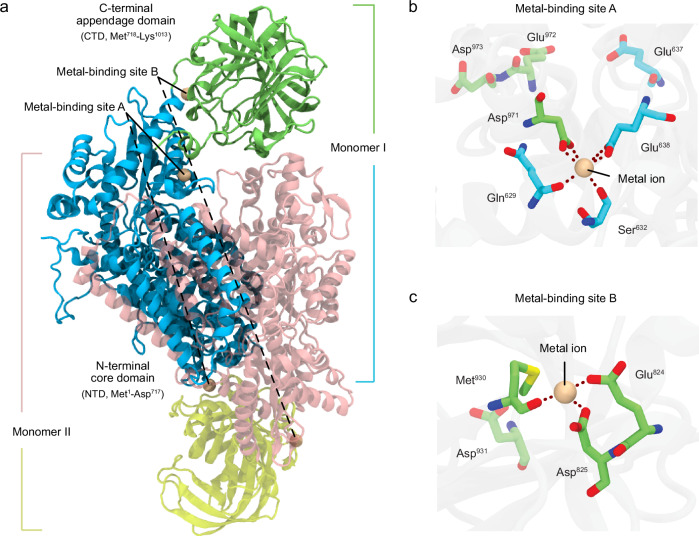
Crystal structure of *Pc*ncAAAD homodimer and metal-binding sites. **a** Structural details of *Pc*ncAAAD homodimer. Each monomer of *Pc*ncAAAD comprises an N-terminal core domain (pink and cyan) that harbors the conserved active structure of PLP-dependent AAADs, along with a unique C-terminal appendage domain (yellow and lime). Close-up of metal-binding site A (**b**) and B (**c**). Residues involved in the two metal-binding sites in the crystal structure are displayed.

*Pc*ncAAAD functions as a homodimer, with its two active sites presented symmetrically at the concealed monomer–monomer interface (Fig. 1a). Each monomer consists of a PLP-dependent catalytic N-terminal domain (NTD, Met1–Asp717) and a non-homologous appendage CTD (Met718–Lys1013). The crystal structure of *Pc*ncAAAD reveals two metal-binding sites occupied by sodium ions within each monomer^6^. Site A resides at the interface between the NTD and CTD, whereas the other site, site B, is located at the interface between two β-barrels within the CTD. Hexa-coordination of an ion is established with protein residues at site A (Fig. 1b), involving two bidentate coordination with the carboxylate of Asp971 from the CTD and the carboxylate of Glu638 from the core NTD, as well as two monodentate coordination with the backbone oxygen of Gln629 and the hydroxyl oxygen of Ser632. In contrast, only tetra-coordination is established at site B (Fig. 1c), comprising one bidentate coordination with the carboxylate of Asp825 and two monodentate coordination with the carboxylate of Glu824 and the backbone oxygen of Met930. Previous experimental studies demonstrated a significant acceleration of the enzyme’s turnover rate when the reaction occurred in a calcium buffer as compared to a sodium buffer^6^. Notably, its activity was completely lost when *Pc*ncAAAD was expressed without the appendage CTD, but partially restored when the truncated enzyme was coexpressed with the CTD. Therefore, the CTD appears to be essential for *Pc*ncAAAD’s catalytic activity, although exactly how this non-catalytic appendage domain enables calcium activation of the enzyme remains elusive.

Calcium exhibits high coordination flexibility, characterized by a coordination number that usually ranges from six to eight but can be as high as 12, with variable bond lengths and angles^11^. Due to its unique coordination chemistry, calcium ligation usually occurs in geometry of a pentagonal bipyramid within protein oxygen atoms and acts as a crucial allosteric regulator in the structural stability, conformational changes and catalytic activities of enzymes^12,13^. For instance, the EF hand motif is a helix-loop-helix calcium-binding motif found in many calcium sensor proteins such as parvalbumin and calmodulin^14^. The EF hand motif in calmodulin binds calcium to induce conformational change that exposes hydrophobic patches on the protein surface, enabling its interaction with target proteins^15,16^. Unlike the EF hand motif, other calcium-binding motifs, for instance the C_2_-domains in protein kinase C (PKC), specifically bind calcium to stabilize its structure. This structural stabilization increases the PKC’s affinity for phosphatidylserine, facilitating the translocation of PKC to the plasma membrane^17^. Elucidating the calcium activation mechanism of *Pc*ncAAAD will not only decipher the specific role played by calcium in this noncanonical aromatic amino acid decarboxylase but also deepen our understanding of calcium-binding and metal-activated enzymes in general and provide critical insights into the design of engineered enzymes for efficient industrial production of aromatic amino acid-derived drugs.

In this work, we first employ microsecond molecular dynamics (MD) simulations with the classical, non-polarizable CHARMM force field^18^ to examine the conformational dynamics of both the full-length *Pc*ncAAAD and its CTD-truncated structure in a sodium and/or a calcium buffer. We then employ a multi-site calcium model^19^ as well as the Drude oscillator^20^, a polarizable model that provides a more accurate description of divalent cations, to complement the classical CHARMM force field simulations in differentiating the binding behaviors of Ca^2+^ and Na^+^. To assess the impact of CTD truncation or ion binding on enzyme stability and activity, we introduce a rating model based on relative conformational variations (RCV), which utilizes a RMSD-based scoring scheme. This model enables us to evaluate the contributions of individual protein segments to the overall structural stability and function of the enzyme. Building upon this framework, we design a series of site-directed mutants to disrupt a key “lid-rim” structure capping the active pocket and use RCV scoring to predict their detrimental effects on enzymatic activity, which are subsequently validated through in vitro assays. Next, we simulate mutants targeting the two metal-binding sites of *Pc*ncAAAD. These simulations and the corresponding enzymatic assays enable us to differentiate the role of the two sites, showing that site A is crucial for maintaining a functional lid-rim structure and enabling the enzyme’s activation, whereas site B plays a supportive role by stabilizing the CTD structure. Finally, through simulating the holo-enzyme in complex with a reaction intermediate, we show that Ca^2+^ is unlikely to participate in direct interactions with the substrate or the PLP co-factor. Overall, our findings provide new insights into the intricate structure–function relationship underlying the calcium activation of *Pc*ncAAAD, while also uncovering generalizable principles for the design and engineering of metal-activatable enzymes for diverse applications.

## Results

### Ca^2+^/Na^+^-induced conformational change

**Fig. 2:**
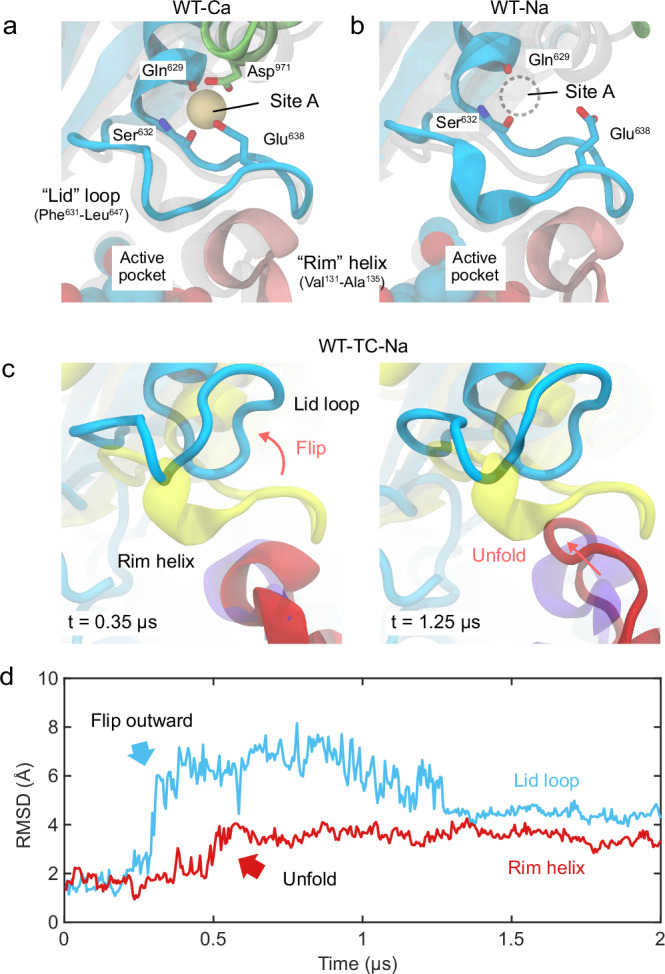
Conformations of the capping lid-rim structure. **a**, **b** Representative snapshots of wild-type *Pc*ncAAAD simulations with calcium (WT-Ca) or sodium (WT-Na). **a** The lid loop (cyan) of site A consistently interacts with the rim helix (pink) of the active pocket in the simulations of WT-Ca, serving as a cap for the active pocket. **b** The lid-rim structure in the simulations of WT-Na and an “empty” site A due to sodium unbinding. **c** Upon the truncation of the CTD, flipping of the lid loop (cyan) induces the unfolding of rim helix (red), as revealed by a simulation of CTD-truncated *Pc*ncAAAD with sodium (WT-TC-Na). **d** RMSD variation of the lid loop and rim helix during the WT-TC-Na simulation shown in (**c**).

We first investigated the conformational variation of a C-terminal-truncated wild-type *Pc*ncAAAD (WT-TC) reportedly to have no decarboxylation activity^6^, in comparison to the full-length wild-type *Pc*ncAAAD (WT) in either calcium (WT-Ca, fully active) or sodium (WT-Na, with reduced activity) buffers. At the interface between the metal-binding site A and the active pocket, a lid-rim structure consisting of a “lid” loop (Phe631–Leu647) and a short helix (Val131–Ala135) at the “rim” of the active pocket reveals distinct conformations for each of these three sets of simulations. Compared to the stable conformation of the lid-rim structure in WT-Ca (Fig. 2a), the loose binding of Na^+^ to site A in WT-Na rendered this site predominantly empty, destabilizing its original conformation (Fig. 2b). Located at the end of the lid loop, a distorted site A could unlock the loop from its original, capping position above the rim helix. This destabilizing effect was most evident upon the truncation of the CTD, where the flipping of the lid loop was followed by the unfolding of the rim helix (Fig. 2c). Collectively, drastic conformational change above the active pocket occurred within this capping structure. The rim helix is part of the active pocket of *Pc*ncAAAD, contributing Val131 and Ala132 to the hydrophobic environment for the aromatic substrates. The unfolded rim helix of WT-TC-Na dislocated the above hydrophobic residues and disrupted the substrate binding environment, which likely led to the eventual loss of enzymatic activity (Fig. 2c–d).

**Fig. 3:**
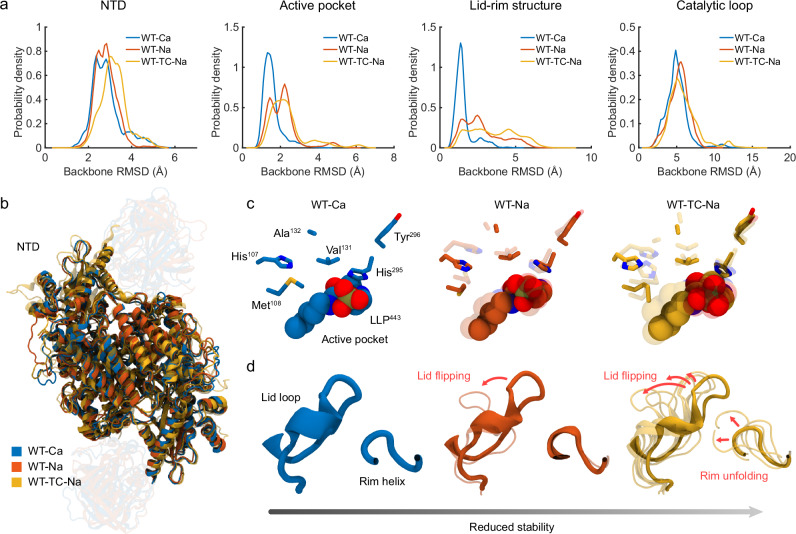
Conformational changes of key structural components in *Pc*ncAAAD. **a** RMSD probability density (Å^–1^) distribution of the NTD, active pocket, lid-rim structure, and catalytic loop, with the first three showing a descending severity in conformational distortion from WT-Ca to WT-Na and then WT-TC-Na. Clustering analysis results reflect a decrease in the structural stability of the NTD (**b**), active pocket (**c**), and lid-rim structures (**d**) from WT-Ca to WT-Na and then WT-TC-Na. The centroid structure of each cluster is rendered by varying transparency, with the top cluster shown as opaque and smaller clusters as transparent. In (**d**), the cartoon thickness is scaled by the relative population of each cluster.

To quantify the calcium and sodium-induced conformational variation of *Pc*ncAAAD, we then determined the backbone RMSD of four potentially activity-correlated components, including the NTD, the active pocket, the lid-rim structure, as well as a catalytic loop essential for AAAD’s decarboxylation activity^21,22^, where WT-TC-Na served as the completely inactive reference (Fig. 3a). Out of the four structural elements, the RMSD of the catalytic loop does not exhibit significant difference between the simulated systems (Fig. 3a and Supplementary Fig. 1). This is consistent with its known high flexibility and a previously reported bidirectional movement among open, closed, and “wide-open” states, all of which can yield comparable RMSD values with respect to the apo-structure^22^. Therefore, we focus on the RMSDs of the remaining three structural elements in our subsequent analysis. The RMSD median and fluctuation of these components largely follow an ascending trend from WT-Ca to WT-Na and then WT-TC-Na (Fig. 3a), reflecting their decreased structural stability. This observation is supported by subsequent clustering analyses of the selected structural component, which reveal broader conformational distributions following the same ascending trend (Fig. 3b–d). The increase in RMSD appears to be most evident in the lid-rim structure. Clustering analysis clearly shows that WT-Ca presents significantly enhanced structural stability compared to WT-Na and WT-TC-Na, whereas the most severe distortion is seen in WT-TC-Na (Fig. 3d). Along with their descending enzymatic activities assayed by experiments^6^, these results highlight the correlation between the structural stability and enzymatic activity of *Pc*ncAAAD. Considering such a structural stability–enzymatic activity correlation and their proximity to the active pocket, we employ the lid-rim structure as one of the crucial indicators for assessing whether and to what degree a mutant’s activity is impaired.

At this point, it is worth briefly explaining the setup of the simulations described above: the co-factor PLP is covalently linked to a lysine residue (Lys443) in apo-*Pc*ncAAAD; the resulting internal aldimine (LLP) has its phosphates positioned near a histidine residue (His442). Based on their pKa values (Supplementary Table 1) predicted by PROPKA^23,24^ and the pH of our experimental assays (pH = 8), we modeled His442 in its protonated state in both monomers and the LLP as –1 and –2 charged in monomer I and II of apo-*Pc*ncAAAD, respectively (see Method for details). The RMSDs and conformations of the NTD, the active site, and the lid-rim structure described earlier were separately determined for monomer I and II (Supplementary Fig. 2). These results from the individual monomers are consistent with their averages shown in Fig. 3, i.e., the WT-Na and WT-TC-Na systems demonstrate reduced stability compared to the WT-Ca system.

In short, we have shown that the enzymatic activity of *Pc*ncAAAD is correlated with the RMSD features of a series of core catalytic structures. Next, taking WT-Ca as the native conformation with its median RMSDs mapped to 0 and WT-TC-Na as a completely inactive conformation with its median RMSDs mapped to 1, we establish a normalized scoring scheme to quantify the relative conformational variation (RCV) of the three aforementioned structural elements in *Pc*ncAAAD mutants. This RCV rating system is designed to facilitate the assessment of the functional role of each component and to guide the design and in silico evaluation of mutant variants. A score comparable to or even greater than that of the WT-TC-Na (RCV = 1) reflects severe structural variation, which, in turn, suggests that the corresponding mutation is likely to destabilize and deactivate the enzyme. After evaluating these mutations in silico, we then verified their impact with enzymatic assays.

### The essential lid-rim capping structure

**Fig. 4:**
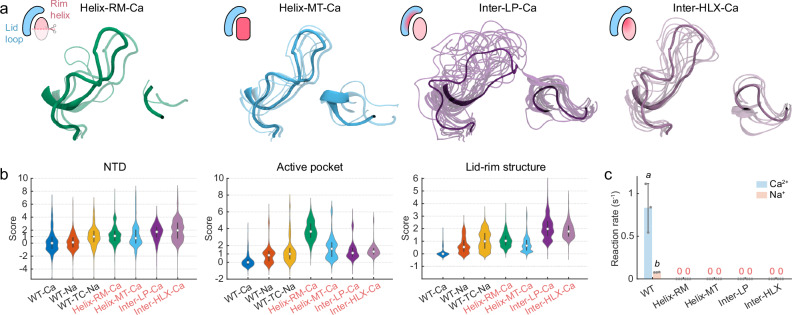
Mutants with disruptive mutations on the capping lid-rim structure. **a** Representative snapshots of the capping lid-rim structure of the mutants with the rim helix destroyed (Helix-RM-Ca: green, Helix-MT-Ca: blue) or the lid-rim hydrophobic interface disrupted (Inter-LP-Ca: purple, Inter-HLX-Ca: puce) revealed by clustering analyses. The nature of the modifications (removal *vs* mutation) is shown by the schematics of each panel. The centroid structure of each cluster is depicted by cartoon representations whose thickness is scaled by relative population, with the top cluster shown as opaque and smaller clusters as transparent. **b** RCV scores. The median values and interquartile ranges are indicated by white markers and black bars, respectively. **c** In vitro enzyme reaction rate in calcium/sodium buffer (*n* = 3). Error bars represent standard deviations, whereas statistically significant differences between non-zero groups (*p* < 0.05) are indicated by italic letters (see Method).

We first designed two mutants with unfolded rim helix, one that directly removed residues Phe133–Ser136 from this helix (Helix-RM-Ca) and the other that unfolded the helical structure by the N130G-S136W-F133G triple mutations (Helix-MT-Ca) (Fig. 4a). With both mutants simulated in a calcium buffer, most of the rating components recorded high scores (median > 1) (Fig. 4b and Supplementary Table 2), demonstrating severe deformation induced in both mutants even under calcium conditions. We therefore hypothesized that the enzymatic function of these two mutants would be severely impaired. This hypothesis was confirmed by subsequent in vitro enzyme assays showing undetectable decarboxylase activity of the two mutants (Fig. 4c). The unfolded rim helix not only destroyed the substrate-binding environment, but also initiated deformation of the entire NTD (Fig. 4b). Notably, the removal or mutation of the rim helix had only a mild impact on the conformational stability of the lid loop (Supplementary Fig. 3b), suggesting a minimal stabilizing effect of the rim helix back on the lid loop.

Given the one-sided stabilizing effect of the lid loop, we hypothesized that this loop assumed a pivotal role in constraining the rim helix through hydrophobic interactions. To validate the role of the lid loop, we designed two more mutants with their hydrophobic interface underneath the lid loop ripped apart by aspartate substitutions on either the lid side (Inter-LP: V633D-P640D-L641D-F646D) or the rim side (Inter-HLX: I97D-P98D-P137D) (Fig. 4a). Although the mutated residues were not near the active pocket, both aspartate mutants disrupted the lid-rim structure and scored comparably to or even higher than the inactive WT-TC-NA for all three rating components (the NTD, the active pocket and the lid-rim structure) in a calcium buffer (Fig. 4a, b and Supplementary Table 2). Consistent with their high RCV scores revealed by these simulations, the enzymatic assays for both Inter-LP-Ca and Inter-HLX-Ca showed undetectable activity (Fig. 4c). Collectively, simulations revealed that the aspartate mutations on either side flipped the lid loop and unfolded the rim helix even in a calcium buffer. These mutations further affected the stability of the entire NTD, and to a lesser extent, the enzyme’s active pocket (Fig. 4b), resulting in its loss of function as confirmed by enzymatic assays. These findings underline the critical role played by lid-rim interface in “gluing” the two monomers through hydrophobic interactions between nonpolar residues on each side: upon the aspartate substitutions, such hydrophobic interactions between the monomers are disrupted, displacing the monomer–monomer interface, which may eventually trigger the disassembly of the homodimer.

Overall, the above results demonstrate that the lid loop maintains the stable conformation of the rim helix in a “capping” manner, ensuring the substrate-binding environment of the active pocket. Therefore, the relative position and conformation of the lid-rim structure can serve as a critical indicator to facilitate the assessment of the structural stability, and, correspondingly, enzymatic activity of a mutant. As revealed in the wild-type crystal structure, Ser632 and Glu638 on the lid loop coordinate the metal ion, serving as a pivotal component of the metal-binding site A. Consequently, calcium binding at site A likely acts to stabilize the lid-rim structure, rationalizing the crucial role of the divalent cation in maintaining the stability of the catalytic structure of *Pc*ncAAAD. In the following section, we further investigate the roles played by *Pc*ncAAAD’s two Ca^2+^-binding sites.

### Site A as the principal metal-binding site

**Fig. 5:**
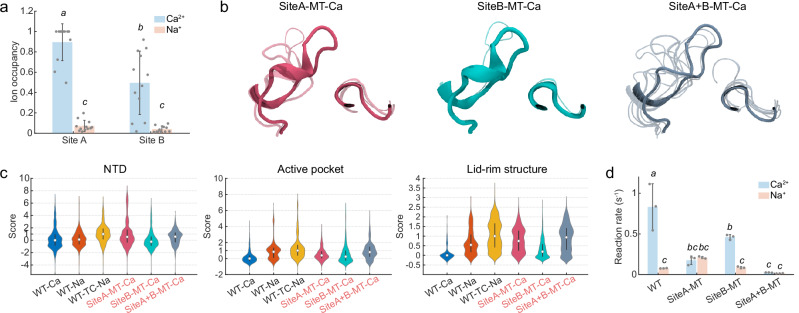
Computationally and experimentally measured stabilization effect of the two metal-binding sites on *Pc*ncAAAD. **a** Ca^2+^/Na^+^ residence at sites A and B during simulations of wild-type *Pc*ncAAAD (*n* = 12; two monomers from six replicas). **b** Representative snapshots of the lid-rim structure in mutants with disrupted site A (SiteA-MT-Ca), site B (SiteB-MT-Ca), or both sites (SiteA+B-MT-Ca) revealed by clustering analyses. The centroid structure of each cluster is depicted by cartoon representations whose thickness is scaled by relative population, with the top cluster shown as opaque and smaller clusters as transparent. **c** RCV scores. The median values and interquartile ranges are indicated by white markers and black bars, respectively. **d** In vitro enzyme reaction rate in calcium or sodium buffer (*n* = 3). Error bars represent standard deviations, whereas statistically significant differences between groups (*p* < 0.05) are indicated by italic letters.

As the wild-type *Pc*ncAAAD crystal structure reveals two metal-binding sites, it was unclear whether calcium activation proceeded through one or both of these sites. To answer this question, we first measured their metal-ion residence probability from the trajectories of WT-Ca and WT-Na (Fig. 5a). During the simulations, a few acidic residues (Glu637, Glu972, Asp973 for site A; Asp931 for site B), apart from the initial ones that provided ion coordination in the crystal structure, stepped in to restore the protein’s calcium coordination when binding with their original partners was transiently lost (Supplementary Fig. 4). Hence, we considered all these “backup” residues together with the initial ones in our subsequent evaluation of the ion residence probability. Overall, Ca^2+^ exhibited 12-fold higher residence probability than Na^+^ at both sites A and B, indicating that they both contributed to *Pc*ncAAAD’s preferential binding towards the divalent cation. (Fig. 5a and Supplementary Fig. 5).

Next, we determined a differential contact map to scan the conformational differences between *Pc*ncAAAD simulations in Ca^2+^ *vs* Na^+^ solutions. Subtracting the residue-based contact map of WT-Ca from that of WT-Na allowed us to identify regions affected the most by differential ion binding (Supplementary Fig. 6): the vast majority of this map has a value close to zero, indicating no significant structural difference; however, two regions clearly stood out, namely, the intra- and the inter-monomer NTD–CTD contact regions, the former of which harbors the metal-binding site A. As discussed earlier, an empty, distorted site A in WT-Na underlies the destabilization of the lid-rim structure and the active site. Additionally, as site A resides at the intra-monomer NTD–CTD interface, its integrity affected the overall NTD–CTD contact: since this site contained three CTD residues (Asp971, Glu972 and Asp973), its distortion significantly weakened the anchorage of the CTD onto the NTD, leaving the former free to detach from the latter (Supplementary Fig. 7). Furthermore, the CTD itself experienced reduced stability in WT-Na, where a “splitting” deformation of its two β-barrels was captured. Albeit to a lesser extent, such deformation was also observed upon the occasional unbinding of Ca^2+^ from site B in WT-Ca. Collectively, these results confirm that Ca^2+^ stabilizes *Pc*ncAAAD and that this effect is primarily mediated by site A through its stabilization of the key lid-rim structure at the intra-monomer NTD–CTD interface, while site B plays a supportive role by stabilizing the CTD and the inter-monomer NTD–CTD contact. The tighter binding of Ca^2+^ than Na^+^ to these two sites maintains the interfacial association between the NTD and the appendage CTD, thereby contributing substantially to the overall structural stability of the protein in the calcium buffer.

The above results obtained with the classical CHARMM force field are corroborated by simulations using a multi-site Ca^2+^ model^19^. Previous studies have shown that this model yielded improved performance in accurately capturing ion–protein binding energies^19^. From six replicas of 1-µs simulations using this multi-site Ca^2+^ model, we found that the Ca^2+^ and Na^+^ residence probabilities were on par with those obtained using the classical CHARMM model (Supplementary Fig. 8). Next, to further evaluate the performance of the classical force fields, we performed a 100-ns polarizable Drude simulation of the full-length *Pc*ncAAAD, during which calcium binding at site A was characterized by ~100% residence probability, in line with the overall high residence probabilities in the initial 100-ns as well as the complete 6-µs CHARMM simulations (100.0% and 71.3%, respectively, see Supplementary Fig. 9). The full-length *Pc*ncAAAD Drude simulations revealed a residence probability of Ca^2+^ at site B (50.7%) comparable to the classical CHARMM (49.5%) simulation of *Pc*ncAAAD. However, unlike in the classical CHARMM simulation, where unbinding of Ca^2+^ from site B was followed by free diffusion of the divalent cation, in Drude simulations, the unbound Ca^2+^ remained associated with Glu824 and Asp825 from one of the two β-barrels of the CTD (the coordination between Ca^2+^ and Met930, Asp931 from the other β-barrel was lost). The polarizable Drude oscillator model has been reported to exhibit a significant enhancement in its ability to describe divalent ion/protein interactions compared to classical additive force fields such as CHARMM C36m^20^. Nonetheless, the large system size of the full-length *Pc*ncAAAD precluded extensive simulations using this polarizable model. Therefore, to thoroughly examine Ca^2+^ binding behaviours at site B, we conducted two replicas of 1-µs Drude simulations of the CTD alone in either Ca^2+^ or Na^+^. These simulations confirmed the preference of site B towards Ca^2+^ (residence probability 81.6%) over Na^+^ (5.3%) and validated the role played by this site in stabilizing the CTD. As shown in Supplementary Fig. 10, upon substitution of Ca^2+^ by Na^+^, the two β-barrels of CTD underwent the same “splitting” deformation recorded earlier in CHARMM simulations (Supplementary Fig. 7). Collectively, these results pinpointed the role of site B in Ca^2+^ binding and CTD stabilization. Next, to further distinguish the significance of the two metal-binding sites in calcium activation of *Pc*ncAAAD, we set out to destroy site A (SiteA-MT-Ca, Asp971, Glu972, Asp973, Glu638, and Glu637), site B (SiteB-MT-Ca, Glu824, Asp825, and Asp931), or both sites (SiteA+B-MT-Ca) with alanine substitutions of the aspartates and glutamates that engaged in the coordination of calcium (Fig. 5b–d and Supplementary Fig. 11).

The site B mutant (SiteB-MT-Ca) exhibited RCV scores comparable to or lower than the WT-Na (Fig. 5c and Supplementary Table 2), suggesting that mutating this site impacted minimally the stability of the core catalytic structures, despite its somewhat underestimated binding affinity by classical CHARMM simulations. In contrast, the alanine substitutions at site A had a direct and significantly greater impact on enzyme structure (Fig. 5c and Supplementary Table 2). In addition to the core structures, severe distortion was also observed in the CTD of SiteA-MT-Ca and SiteA+B-MT-Ca (Supplementary Fig. 12). Based on the above findings, we hypothesize that site A is the primary metal-binding site in mediating the calcium activation of *Pc*ncAAAD, whereas site B may play a subtle, auxiliary role. Such a prominent role of site A can be explained by its interfacial location, where the nearly constantly bound Ca^2+^ stabilizes the negatively charged interface between the NTD and the CTD, preserving the structural integrity of the enzyme’s active site.

The in vitro enzyme reactions support our hypothesis that site A is more important for the catalysis and calcium activation of *Pc*ncAAAD than site B (Fig. 5d). Wild-type *Pc*ncAAAD (WT) has a k_cat_ of 0.51 s^−1^ and a K_m_ of 88 µM in calcium buffer compared to a k_cat_ of 0.074 s^−1^ and K_m_ of 64 µM in sodium buffer, i.e. the enzyme activity is increased by approximately 7-fold and the catalytic efficiency (k_cat_/K_m_) is increased 5-fold upon calcium binding. (Table 1). The mutant with site B destroyed (SiteB-MT) has a slightly reduced k_cat_ at 0.41 s^−1^ in calcium buffer and 0.059 s^−1^ in sodium buffer, and the effect of calcium activation is slightly affected compared to WT. In contrast, the enzyme with mutations to destroy calcium-binding at site A, SiteA-MT, has a dramatically reduced k_cat_ at 0.073 s^−1^ in calcium buffer, along with a k_cat_ of 0.038 s^−1^ in sodium buffer, reflecting its significantly reduced catalytic performance and calcium activatability upon the disruption of site A. Overall, while mutating either site A or site B impairs catalysis, the effect is much more pronounced for SiteA-MT, which also demonstrates significantly reduced calcium activation.

**Table 1:** Kinetic properties of *Pc*ncAAAD mutants. Kinetic parameters obtained by fitting Michaelis–Menten model via non-linear regression in GraphPad Prism v 8.4.3

	k_cat_, s^−1^		K_m_, µM		Fold activation (k_cat_, Ca/Na)	Fold catalytic efficiency (k_cat_/K_m_, Ca/Na), µM^−1^s^−1^
	Calcium	Sodium	Calcium	Sodium		
WT	0.51 [0.45–0.58]	0.074 [0.068–0.079]	88 [49–154]	64 [45–89]	6.9x	5.0x
SiteA-MT	0.073 [0.061–0.089]	0.038 [0.032–0.047]	513 [319–839]	332 [184–619]	1.9x	1.2x
SiteB-MT	0.41 [0.36–0.48]	0.059 [0.054–0.064]	174 [105–287]	87 [60–126]	6.9x	3.5x
SiteA+B-MT	0.044 [0.037–0.055]	0.026 [0.022–0.031]	586 [359–979]	527 [336–843]	1.7x	1.5x

Values in the brackets represent 95% confidence intervals. (See Supplementary Fig. 13 for raw kinetics data).

The role of site B can be further understood through two other pieces of evidence. First, site A and site B mutations seem to exert an additive effect on *Pc*ncAAAD k_cat_. SiteA+B-MT has a k_cat_ of 0.044 s^−1^ in calcium buffer and 0.026 s^−1^ in sodium buffer. The slightly reduced activity of SiteA+B-MT in both calcium and sodium buffer compared to SiteA-MT suggests that mutations at site B further decrease enzymatic activity in an additive manner to the mutations at site A. Second, the melting curves of *Pc*ncAAAD and the corresponding mutants shed light on the role played by site B in calcium-mediated stabilization of the enzyme. WT *Pc*ncAAAD has three different melting temperatures (T_m_), which may correspond to the unfolding of different domains. Taking the deepest well in the derivative graph to be the primary T_m_ for each enzyme (Supplementary Fig. 14), all mutations at sites A and B are found to decrease the structural stability of *Pc*ncAAAD, as evidenced by the decreased T_m_ of SiteA-MT, SiteB-MT, and SiteA+B-MT compared to WT (Supplementary Table 3). The melting curve for the WT shows a rightward shift (increased by 1.8 °C) from sodium to calcium buffer, indicating that the wild-type enzyme is stabilized by calcium binding (Supplementary Fig. 14, Supplementary Table 3). Interestingly, SiteA-MT exhibits this rightward shift in melting temperature in response to calcium (+2.3 °C), but SiteB-MT and SiteA+B-MT do not. These results suggest that only with an intact site B, the enzyme exhibits increased structural stability upon calcium binding. This is consistent with the role of site B in calcium-mediated stabilization of the CTD, which reduces the latter’s large-scale detaching and flipping motions (Supplementary Fig. 7). Curiously, SiteB-MT and SiteA+B-MT exhibit a leftward shift in T_m_ of 1.5 and 1.7 °C, respectively, upon switching from a sodium to a calcium buffer. Given that significant deformation is recorded at the NTD in siteB mutant simulations and that SiteB-MT retains its calcium activatability, these results suggest that mutation at site B alters the thermal response of *Pc*ncAAAD towards different ionic environments and while the nature and mechanism of this altered thermal response remains unclear, the non-catalytic domains of the enzyme, such as the NTD is likely involved. In summary, we conclude that site B stabilizes the CTD of *Pc*ncAAAD, thereby contributing to its overall structural stability, while site A helps maintain the local structure of its active site, and, unlike site B, is directly responsible for the calcium activation behavior of this enzyme.

### Absence of Ca^2+^ interaction with substrate in holo-*Pc*ncAAAD

Notably, the 3D ion occupancy analysis of apo-*Pc*ncAAAD simulations shows that Ca^2+^ did not enter its active site in any of the six 1-µs simulations. In contrast, the presence of Na^+^ in the enzyme active site is clearly captured (Supplementary Fig. 15). It therefore appears that the reduced stability of the enzyme in Na^+^ solution may have increased the ion accessibility of its active site. However, as these simulations were all conducted using the crystal structure of *Pc*ncAAAD solved in the apo state, whether Ca^2+^ may directly contact and interact with the substrate remains unclear.

**Fig. 6:**
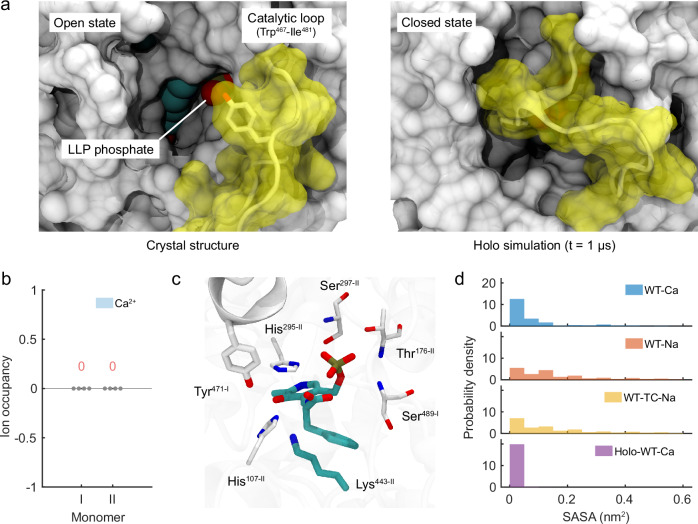
MD simulations of holo-form WT-Ca with external aldimine (Holo-WT-Ca). **a** Comparison of the initial crystal structure and the final snapshot of 1-µs holo simulation, illustrating the closure of a large catalytic loop over the active pocket. **b** Ion occupancy within the active pocket during the simulations (*n* = 4). **c** Close-up of the active pocket of monomer II in the final structure (*t* = 1 µs). **d** Probability density (nm^–2^) histogram of the solvent-accessible surface area (SASA) for the PLP phosphate group of monomer II.

To address this question, we constructed a holo-*Pc*ncAAAD by introducing a reaction intermediate, namely, the external aldimine, into its active site. However, the enzyme’s transition from an open to a closed state typically occurs beyond the microsecond timescale^21,22^, precluding it from being captured by plain MD simulations. Therefore, we superimposed *Pc*ncAAAD onto its plant counterpart, *Ps*TyDC. Based on the closed-state structure of the latter enzyme, we identified the reference location of a key catalytic tyrosine and then applied a harmonic restraint to force *Pc*ncAAAD switching from the open to the closed state (see Method). Finally, we conducted four replicas of 1-µs simulations to allow the catalytic loop harbouring Tyr471 to fully relax. These simulations consistently produced the closed-state loop conformation of *Pc*ncAAAD in one monomer (Supplementary Fig. 16a, b). In the other monomer, a Ca^2+^ ion initially trapped near Glu353 at the active-site entrance displaced the catalytic tyrosine, failing to maintain the loop in its closed state (Supplementary Fig. 16c). No Ca^2+^ was found inside the active site of either monomer of *Pc*ncAAAD in its four simulation replicas (Fig. 6b). Analysis of the active pocket capped by the closed-state loop in these trajectories indicates that the external aldimine was tightly sealed (Fig. 6a). In such a closed pocket, the aromatic ring of the external aldimine was buried deeply inside the active site with few nearby water molecules, where the presence of a Ca^2+^ ion would introduce significant steric clashes with active-site residues (Fig. 6c). The phosphates of the external aldimine demonstrated significantly reduced solvent accessible surface area (SASA) in holo-*Pc*ncAAAD compared to those of the internal aldimine in the open-state, apo-enzyme (Fig. 6d). Collectively, these simulations indicate that the large catalytic loop of the enzyme forms an overall tight seal of its active site, precluding the divalent cation from entering. As a result, Ca^2+^ interacts with neither the deeply buried aromatic ring of the substrate nor the well coordinated phosphates of LLP. These findings strongly suggest that Ca^2+^ is unlikely to participate in direct interaction with either the substrate or the PLP co-factor of *Pc*ncAAAD.

## Discussion

As a type-II PLP-dependent decarboxylase sharing considerable structural similarities with many plant AAADs, *Pc*ncAAAD has an active pocket sandwiched between its catalytic loop and the cofactor PLP, which provides the structural basis for the binding and decarboxylation of aromatic amino acids^21^. Unlike plant AAADs, *Pc*ncAAAD possesses two metal-binding sites, one of which (site A) sits right above a short helix that caps the enzyme’s active site. The fungal decarboxylase additionally contains an appendage CTD that harbors a second metal-binding site (site B). Relative to a sodium buffer, *Pc*ncAAAD in a calcium buffer demonstrates significant increase in catalytic efficiency. Combining molecular dynamics simulations, in vitro enzyme reactions, and protein stability measurements, our study has highlighted the atomistic details and mechanism underlying such calcium activation of *Pc*ncAAAD. Located at the intra-monomer NTD–CTD interface, the hexa-coordination of divalent Ca^2+^ at the site A of *Pc*ncAAAD “glues” the NTD and CTD domains to retain an overall structural integrity of the enzyme. Furthermore, calcium binding at site A stabilizes the coordination of a 17-amino-acid lid loop (Phe631–Leu647) in a lid-rim structure capping the active site. Supported by both simulation and experimental results of destructive mutants, we have shown that this lid-rim capping structure is essential to *Pc*ncAAAD’s activity, and its deformation captured by simulations could be used as a predictor of enzyme deactivation. Along this line, the experimentally demonstrated inactivity of WT-TC-Na has been linked to its destroyed site A, inducing a series of drastic conformational variations that include the severe deformation of the lid-rim structure.

Building upon the above simulation and experimental findings, we have developed a RMSD-based RSV score to evaluate the degree of conformational change of key structural components in a given *Pc*ncAAAD mutant. By combining this scoring scheme applied on the in silico models with the experimental assessment of enzyme reaction kinetics and thermal stability, we further dissect the impact of the two calcium-binding sites in *Pc*ncAAAD. We show that site A is the primary site of action responsible for the conformational stabilization and significantly increases enzymatic activity upon calcium binding. In contrast, we present that site B plays a weaker but nonetheless indispensable role in promoting the enzyme’s calcium-mediated structural stabilization. Site B resides at the interface between the double β-barrels of the CTD, a region that lacks strong inherent packing interactions and is rich in acidic residues such as Glu824, Asp825, and Asp931. The presence of the divalent cation at this site forms a strong linker between the two β-barrels through favorable electrostatic interactions, preventing their otherwise drastic separation occasionally recorded upon Ca^2+^ unbinding. The stable double-β-barrel-fold conformation of CTD is important because it provides the necessary structural stability and facilitates the suitable interacting geometry for two binding interfacial regions, i.e., the inter- and intra-monomer NTD–CTD contact regions (each formed by one of the two β-barrels), ultimately promoting the integrity of the NTD–CTD interface. The disruption of site B reduces structural stability but does not fully abolish activity, which may be attributed to the swinging motion of the CTD allowing for intermittent re-establishment of a functional NTD-CTD interface. Collectively, our findings reveal that calcium activates *Pc*ncAAAD primarily in a structure-related way, i.e., through stabilizing the enzyme’s active pocket upon binding to site A and enhancing the structural integrity of the CTD upon binding to site B. While the non-catalytic CTD is distant from the enzyme active site, it contributes three acidic residues (Asp971, Glu972, and Asp973) forming part of the site A, which underlies the inactivity of *Pc*ncAAAD upon CTD truncation and its partially restored activity upon coexpression with the CTD^6^.

All our in silico models for the WT and mutants were simulated using the CHARMM36m force field, which models the divalent calcium ion as a fixed-charge, non-polarizable hard sphere. We validated the classical model’s performance for the metal-binding site by implementing two fine-tuned models reportedly to be more accurate in specific scenarios: a multi-site model, which incorporates charge dispersion across dummy sites, and the Drude polarizable model, which explicitly accounts for electronic polarizability. The Drude polarizable simulations yielded Ca^2+^ occupancy at the metal-binding sites comparable to those from the classical CHARMM36m simulations, supporting the plausible use of the classical model for this rating system. The multi-site model exhibited a somewhat lower Ca^2+^ occupancy and a higher exchange frequency. This difference may arise from the multi-site model being tuned for systems requiring a less “sticky” Ca^2+^ representation (e.g., permeation kinetics studies). The overall consistency between the Drude and classical CHARMM36m model validates the latter’s description of Ca^2+^ binding within our system. However, given that conformational changes in a large protein, such as *Pc*ncAAAD, may occur on a timescale longer than the microsecond simulations performed here, we cannot rule out the possibility that the sampled conformations are not fully converged. Furthermore, in silico mutations pose an interpretive challenge as they may induce unintended destabilization of the overall protein fold. For instance, the S136W mutation in the Helix-MT mutant, designed to locally disrupt the rim helical structure via the bulkier tryptophan side chain, may instead trigger a global destabilizing effect on the overall protein structure, potentially leading to a loss of enzyme activity through an off-target structural mechanism. For the above reasons, the RCV scores computed from simulations are used to assess loss-of-function mutations qualitatively and should not be interpreted as a quantitative predictor for enzymatic activity.

Guided by insights from in silico models, we have conducted validation through mutagenesis experiments on selected mutation candidates, ensuring the confidence of the proposed mechanism. We should also add that the crystal structure of *Pc*ncAAAD is solved in an open, apo conformation instead of a closed, holo one, which precludes straightforward analysis of the impact of calcium on substrate binding. However, Dunathan’s hypothesis predicts that the decarboxylation reaction would be significantly inhibited by metal ions due to the metal–carboxylate binding^25^. Our simulations of apo WT systems demonstrated a clear distinction: the active site was not visited by Ca^2+^, but was susceptible to Na^+^ intrusion. We infer that Ca^2+^ binding exerts a stabilizing effect on the overall protein structure, which is crucial for limiting ion access to the catalytic center. Furthermore, our holoenzyme simulations demonstrated the complete sealing of the active site upon catalytic loop closure, which effectively occludes the pathway for ion diffusion from the bulk solution. This reflects the tight control exerted by the enzyme to prevent interfering ions from entering into the catalytic center, while it tunes the local dielectric environment to favor the electrostatic interactions of the PLP-substrate Schiff base. Based on these evidences and the at least 22-Å distance between the PLP cofactor and the two calcium binding sites, the calcium-activation mechanism of *Pc*ncAAAD is unlikely to involve direct interactions between the divalent cation and the substrate. Within the remaining framework of calcium–protein interactions, the RSV score developed here serves as a straightforward metric for assessing the stability and loss-of-function mutations of *Pc*ncAAAD, which may be readily extended and employed in the study of structure-related activation of other metal-binding enzymes.

The impact of calcium ions on protein structure and function is typically conveyed through three distinct mechanisms: indirect regulatory, direct catalytic, or direct structural^26^. Intracellular proteins such as troponin C and calmodulin are known to undergo conformational changes upon calcium binding^14,27^. These proteins then transmit their altered conformations to a multitude of proteins they bind, thereby enabling calcium signals to regulate a wide range of cellular activities. Alternatively, calcium can play a direct catalytic role, such as in phospholipase A2 and phosphoinositide phospholipase C^28,29^. In these enzymes, calcium ion resides within the active site and acts as a Lewis acid to stabilize transition states or orient substrates correctly for the reaction to occur. Conversely, other proteins, including eukaryotic endoribonucleases and proteolytic enzymes such as certain serine proteases, directly bind calcium ions for structural stabilization^26,30^. In these enzymes, the divalent cation tends to reside within surface loop regions distal to the catalytic residues, where they are believed to play mostly a stabilizing role. Furthermore, some enzymes bearing multiple calcium binding sites are activated via a “mixed” mechanism, such as thermolysin-like proteases and α-amylases^31,32^. Our findings of *Pc*ncAAAD suggest that its calcium activation falls in the category of direct structural stabilization rather than catalytic participation. We have shown that the two metal-binding sites in *Pc*ncAAAD both play a part in a stabilizing mechanism to activate the enzyme. Specifically, site A serves as a focal point for intra-monomer coordination, anchoring the CTD to the catalytic NTD and ensuring the integrity of the lid-rim structure; Site B stabilizes the CTD’s double β-barrel fold, preventing the splitting or detaching that would otherwise compromise the interfacial geometry required for site A. This hierarchical stabilization highlights a sophisticated regulatory architecture that enables a distal, non-catalytic domain, i.e., the CTD, to deliver a substantial impact on the activity of the enzyme.

In the specific context of PLP-dependent decarboxylases, our discovery of this dual-site calcium activation mechanism expands the understanding of AAAD structural diversity. It suggests that the fungal lineage of these enzymes may have evolved the CTD not merely as a structural appendage, but as a responsive regulatory element that utilizes environmental calcium ions to fine-tune catalytic readiness. This indirect “metal-stabilization” mechanism has offered a transferable, versatile strategy for enzyme engineering of other industrially relevant AAADs. Ultimately, our in silico model and RSV scoring system provide a framework for investigating how distal metal-binding events can be transduced into activation in complex metalloprotein systems.

In conclusion, our work presented here offers mechanistic insights into the calcium activation of a noncanonical fungal aromatic L-amino acid decarboxylase. Employing an RMSD-based scoring scheme tailored for the enzyme, we assess a series of carefully designed *Pc*ncAAAD mutants through subunit structural dynamics, followed by experimental validation. Our findings reveal that site A, located at the intra-monomer interface between the NTD and the CTD, is crucial for the enzyme’s activation, whereas site B has a secondary, supportive function. Calcium binding at site A mediates conformational stabilization of a protective lid loop, preserving the conformation of a helix at the rim of the active pocket, which is essential for substrate binding. Disruption of site A, either through mutations or removal of the CTD, leads to deformation of the lid loop and subsequent enzyme deactivation, despite the presence of intact catalytic structures. In addition, calcium binding at site B stabilizes the double β-barrels of the CTD, promoting the structural and functional integrity of the NTD–CTD interface. Furthermore, the closure of the catalytic loop in holo-*Pc*ncAAAD seals the active site, preventing ion intrusion and supporting the above structural activation mechanism over the direct participation of calcium in the catalytic process. With combined theoretical and experimental evidence, our study reveals a previously unrecognized noncanonical mechanism of action for the calcium-activatable *Pc*ncAAAD and lays the foundation for future studies on the rational design and engineering of metal-binding enzymes with improved stability and enhanced activity.

## Method

### Protein structure preparation

Crystal structure of the *Pc*ncAAAD dimer was originally solved in a previous study^6^ (PDB code 6ebn). The structure of a missing loop (Ile^765^–Arg^770^) was predicted using the Modloop server^33,34^ and merged into the solved structure. This resulting structure is referred to as the full-length, wild type (WT) *Pc*ncAAAD structure. To investigate the function of the appendage CTD, a truncated structure of *Pc*ncAAAD without the appendage CTD was adopted, namely WT-TC. All analyses for WT and WT-TC systems were conducted on both monomers, with results for each individual monomer provided in the Supplementary Information. The WT *Pcnc*AAAD structure was employed as a homology template to predict the structures of mutants using Modeller v10.7^35,36^ as listed in Table 2.

**Table 2:** Mutant list

System	Ion condition	Mutations
Helix-RM-Ca	0.05 M CaCl_2_	Remove F133–S136
Helix-MT-Ca	0.05 M CaCl_2_	N130G, S136W, F133G
Inter-LP-Ca	0.05 M CaCl_2_	V633D, P640D, L641D, F646D
Inter-HLX-Ca	0.05 M CaCl_2_	I97D, P98D, P137D
SiteA-MT-Ca	0.05 M CaCl_2_	E637A, E638A, D971A, E972A, D973A
SiteB-MT-Ca	0.05 M CaCl_2_	E824A, D825A, D931A
SiteA+B-MT-Ca	0.05 M CaCl_2_	E637A, E638A, D971A, E972A, D973A, E824A, D825A, D931A

### MD simulations of WT and mutant *Pc*ncAAAD

Unless otherwise noted, all MD simulations were carried out using GROMACS 2024.5^37^ with classic CHARMM36m force field^18^ and CHARMM General force field (CGenFF)^38–40^. Based on PROPKA^23,24^ predictions (Supplementary Table 1), we estimate that the residue His442 is overwhelmingly (>95%) protonated (+1 charged). Therefore, this residue was modeled in the +1 charged state for both monomers in apo-*Pc*ncAAAD; As for LLP, given that its second pKa is around 7 and that PROPKA estimates have an uncertainty of 0.65 to 1.00 pH unit^24^, both the −1 and −2 charged states of LLP likely have a significant population under the experimental condition (pH = 8). Given the large distance between LLPs from the two *Pc*ncAAAD monomers (~18 Å), their protonation states are unlikely to depend on each other. Therefore, to efficiently study both states, we modelled LLP as −1 and −2 charged in monomer I and II, respectively, consistent with the trend revealed by their pKa estimates (Supplementary Table 1). The LLP cofactors, in both its −1 and −2 protonation states, were parameterized using the CGenFF website interface. Their force field parameters, along with other simulation input/output files, are provided on Figshare (see “Data availability”). Topology and parameters for the multi-site calcium ion model (CAM) were adopted from the previously published work by Song et al.^19^.

All the simulation systems for wild-type *Pc*ncAAAD and mutants were constructed with the full-length dimeric structure and solvated in a dodecahedron water box. The simulations of WT systems were separately performed in two ion conditions, with 0.05 M CaCl_2_ or 0.05 M NaCl, respectively, whereas the WT-TC system was ionized with 0.05 M NaCl. The mutant *Pc*ncAAAD simulation systems were ionized by 0.05 M CaCl_2_. All systems were ionized to have a neutral charge. Four of the Ca^2+^/Na^+^ ions were initially placed at the metal-binding sites revealed in the crystal structure.

After energy minimization, all the systems successively underwent a 1-ns NVT and a 1-ns NPT equilibration, respectively. Production simulations were carried out in an NPT ensemble. The system temperature was coupled to 300 K using velocity rescaling^41^, whereas the system pressure was coupled to 1 bar using the Berendsen method^42^. Bonds involving hydrogen atoms were constrained using the LINCS algorithm^43^. Non-bonded interactions were cut off at 9 Å, and the particle mesh Ewald (PME) method^44^ was applied for electrostatic interactions. 1-µs production simulations were performed per replica per system. Simulations for WT-Ca (including the systems using the CAM model), WT-Na and WT-TC-Na were carried out in six replicas, while simulations for all the other systems were conducted in four replicas.

### Polarizable MD simulations using the Drude model

All systems involving the Drude force field^45^ were set up using the Solution Builder and Drude Prepper^46^ on CHARMM-GUI web interface^47,48^ and adapted to NAMD3^49^. Due to the substantial computational cost of Drude simulation, the CTD segment of monomer I (Met718–Thr969) was employed to evaluate the ion occupancy at site B for comparison with classical CHARMM36m simulations; Separately, a full-length *Pc*ncAAAD (with its LLP443 substituted by a lysine) was also subjected to a 100-ns Drude simulation. In both Drude simulations, the protein was placed in the center of a rectangular box and then solvated with four-point polarizable water described by the SWM4-DP model^45^. Either 0.05 M NaCl or CaCl_2_ was added to neutralize the system’s charge and ensure consistent ion conditions with the classical CHARMM36m simulations.

After their construction by the CHARMM-GUI web interface, each of the Drude systems was successively subjected to a 200-step energy minimization and a 100-ps NPT equilibration. Thereafter, two 1-µs production simulations were each carried out for the CTD segment simulations with calcium and sodium, respectively, whereas one 100-ns production simulation was carried out for the full-length *Pc*ncAAAD simulation.

### Modeling and simulations of holo-form *Pc*ncAAAD

Given that the enzyme likely stabilizes the reaction intermediate more strongly than the substrate alone, the holo-*Pc*ncAAAD system was modeled with a reaction intermediate, namely, the enolimine-form external aldimine of phenylalanine, which was parameterized using the CGenFF interface. The holo-form wild-type *Pc*ncAAAD (Holo-WT) structure was generated using a two-step superposition method (Supplementary Fig. 17): 1) The external aldimine was docked by aligning its pyridine ring to the corresponding part of LLP443 in *Pc*ncAAAD (Supplementary Fig. 17a). Subsequently, the PLP part was directly removed from LLP443, and the residue was renamed as lysine. 2) The resulting structure was aligned to the functionally similar PLP-dependent enzyme *Ps*TyDC (PDB code 6eem) using the MultiSeq plugin^50^ of VMD^51^ (Supplementary Fig. 17b). The final structure was solvated, ionized, and subjected to a 100-ns simulation. Similar system configuration and ion conditions in the WT-Ca simulations were applied to this holo-system with additional positional restraints applied to the heavy atoms of substrate, and the Cα atoms of the protein’s secondary structures and the catalytic tyrosine (Tyr471). To guide the closure of the catalytic loop, the Cα reference position for Tyr471 was defined by the Cα position of the catalytic tyrosine (Tyr350) on the catalytic loop of the aligned closed-form *Ps*TyDC (Supplementary Fig. 17c). Finally, four replicas of 1-µs simulations were conducted for the modeled Holo-WT-Ca system with the same positional restraints except for the catalytic loop, which was allowed to move freely.

### Post-simulation analysis and scoring scheme

Ion residence was measured by the presence of ions close to the surrounding residues involved in the binding site, where all potential ion-binding residues near sites A and B were considered. Conformational changes of *Pc*ncAAAD and its subunits were quantified by the backbone RMSD with corresponding parts in the crystal structure as references. Clustering analyses were carried out for each subunit using the GROMOS method^52^. Unless otherwise specified, the RMSD calculation and clustering analysis for a given system utilized both monomers from all replica trajectories, with the structures aligned by their dimeric core NTD. The cutoff values were obtained from the clustering analysis of the WT-Ca trajectories for NTD (2.4 Å), active pocket (1.4 Å), catalytic loop (3.0 Å), lid-rim structure (1.8 Å), separately. To quantitatively inspect how each subunit in the mutants was changed and to what extent these deformations might lead to deactivation, the relative conformational variation of the subunits was measured by the RCV score. In this scoring scheme, we employed the WT-Ca as the active (score = 0.0) and the WT-TC-Na as a fully inactive reference (score = 1.0). All presented structures were visualized and rendered using VMD^51^.

### Plasmid construction

PCR-based mutagenesis using mutational primers was used to generate *Pc*ncAAAD mutant sequences. The template for mutagenesis was the wild-type *Pc*ncAAAD sequence generated previously^6^. Gibson assembly was used to clone the mutant sequences into the bacterial expression vector pHis8-4.

### Protein expression and purification

BL21-(DE3) *E. coli* containing the expression constructs were grown at 37 °C in terrific broth to an OD_600_ of 0.8, induced with 0.15 mM isopropyl-β-D-thiogalactoside (IPTG), and grown at 18 °C for 20 h. The bacterial cells were harvested by centrifugation and resuspended in 100 mL lysis buffer (50 mM Tris [pH 8.0], 0.5 M NaCl, 20 mM imidazole, 50 µM pyridoxal 5’-phosphate (PLP), 1 mg/mL lysozyme, 1 mM phenylmethylsulfonyl fluoride and 1 mM dithiothreitol (DTT)). Resuspended cells were lysed with multiple passes through an M-110L microfluidizer (Microfluidics). The resulting crude protein lysate was clarified by centrifugation prior to Qiagen nickel-nitrilotriacetic acid (Ni-NTA) gravity flow chromatographic purification. After passing the clarified lysate through the column, the resin was washed with 5 column volumes of water followed by 5 column volumes of lysis buffer and the clarified lysate was passed through the column. The column was washed with 5 column volumes of lysis buffer, followed by 2 column volumes of wash buffers 1–3 (50 mM Tris [pH 8.0], 0.5 M NaCl, 50 μM PLP, 1 mM DTT and 50, 75, or 100 mM imidazole for wash buffers 1–3, respectively). Lastly, protein was eluted with 2 column volumes of elution buffer (wash buffer with 250 mM imidazole). His-tagged tobacco etch virus (TEV) protease was added to the eluted protein, followed by dialysis at 4 °C for 16 h in dialysis buffer (25 mM Tris [pH 8.0], 0.1 M NaCl, 50 μM PLP, 0.5 mM DTT, and 5% glycerol). After dialysis, the protein solution was passed through Ni-NTA resin to remove the cleaved tag, the uncleaved recombinant protein, and His-tagged TEV. Prior to freezing, the *Pc*ncAAAD recombinant enzyme was concentrated and buffer exchanged in a 30 kDa amicon filter with storage buffer (20 mM Tris [pH 8.0], 25 mM NaCl, 50 μM PLP, 0.5 mM DTT). The purity and concentration of the recombinant proteins was evaluated by ImageJ densitometric analysis (see Supplementary Figs. 18, 19). SDS-PAGE gels (ExpressPlus PAGE gel, Genscript) were run in MOPS buffer at 125 V for 20 min, then switched to 150 V until the dye front reached the edge of the gel. Gels in Supplementary Figs. 18, 19a were stained with Coomassie Brilliant Blue G-250 dye in 50% methanol 10% glacial acetic acid for 30 min and destained in 50% methanol 10% glacial acetic acid for 1 h, followed by destaining with water overnight. Gel in Supplementary Fig. 19b was stained with InstantBlue Coomassie Protein Stain (ISB1L, Sigma Aldrich) dye for approximately 15 minutes and destained with water.

### Enzyme assays

Frozen protein aliquots were thawed on ice and washed three times in chelation buffer (5 mM 18-crown-6 ether, 5 mM EDTA disodium dihydrate, 50 mM Tris [pH 8.0]) followed by three washes in wash buffer (50 mM Tris [pH 8.0]) to remove metal ions. Washes were performed by diluting enzyme 1:20 in buffer and concentrating in a 50 kDa Amicon Ultra 0.5 centrifugal filter device by centrifugation for 10 mins at 14,000 x *g*, performed at 4 °C. Protein concentration was estimated using A280 on the nanodrop, using extinction coefficients and molecular weight parameters calculated for each mutant. Enzyme assays (both activity assays and Michaelis–Menten kinetic assays) were performed in 100 μL of reaction buffer (50 mM Tris, pH 8.0) containing either 30 mM NaCl or 30 mM calcium acetate. The enzyme concentration was 30 nM (calcium buffer reactions) or 150 nM (sodium buffer reactions). Activity assay reactions were performed at 2 mM L-phenylalanine concentration at room temperature for 10 minutes and quenched with 100 µL 1 M formic acid. Michaelis–Menten kinetic assays were incubated with a range of L-phenylalanine substrate concentrations (1 μM to 2 mM) at room temperature for 10 min and quenched with 100 µL 1 M formic acid. The reaction mixture was centrifuged and the supernatant was analyzed by liquid chromatography-mass spectrometry (LC − MS).

### LC–MS analysis

2 μL of reaction mixture was analyzed by a Vanquish liquid chromatography system (Thermo Fisher Scientific) equipped with a 150 mm C18 column (Kinetex 2.6 μm silica core−shell C18 100 Å pore, Phenomenex), and coupled to an Orbitrap Exploris 120 mass spectrometer (Thermo Fisher Scientific). Compounds were separated by reversed-phase chromatography with a ramp gradient of solvent A (0.1% formic acid in water) and solvent B (0.1% formic acid in acetonitrile): 5% solvent B from 0–2 min, 5−12.5% solvent B from 2–5 min, 95% solvent B from 5–9 min, followed by a final equilibration of 5% solvent B from 9–13 min with a flow rate at 0.5 mL/min. Mass spec was run in positive mode at 3500 V with a scan range from 50 to 200 m/z. Phenylethylamine (PEA) was quantified by extracted ion chromatogram at *m/z* = 122.0964 with 5 ppm mass tolerance (Supplementary Fig. 20). Peak areas calculated on Freestyle software (Thermo Fisher Scientific). PEA production was quantified against a standard curve of analytical standards. Kinetic constants k_cat_ and K_m_ were determined by fitting raw data to the Michaelis−Menten equation using the nonlinear regression function in Prism v. 8.4.3.

### ThermoFluor assays

Thermal stability of wild-type *Pc*ncAAAD and mutants was determined using a fluorescence-based thermal stability assay. In individual wells of a 384-well plate, 2.5 μg of enzyme was mixed with 2 μL 50x-diluted SYPRO Orange dye and 7 μL buffer to a total volume of 10 μL. Buffer was 50 mM Tris, pH 8.0 and contained either 30 mM NaCl or 30 mM calcium acetate. Assay was performed in a LightCycler 480 II real-time PCR machine (Roche). The plate was heated from 20 °C to 85 °C with a heating rate of 0.06 °C/s. Fluorescence intensity was measured with Ex/Em: 465/580 nm. Protein melting points (T_m_) were calculated from the derivative of the melt curve by LightCycler 480 Software release 1.5.1.62 SP2.

### Statistics and reproducibility

Sample groups with zero variance (all values being zero) were excluded from comparative analyses to prevent the over-correction of *p*-values. For comparisons between two independent groups, a two-tailed unpaired Student’s *t*-test was employed. For multiple group comparisons where each group has at least three samples, a one-way analysis of variance (ANOVA) followed by Tukey’s post-hoc test for multiple comparisons was used. All statistical tests and analyses were conducted using Prism 10 (GraphPad). MD simulations of each system were performed at least four times independently, and the mean value of each replica is presented; each in vitro experiment was conducted three times independently, with all individual data points presented. Statistical significance was defined as *p* < 0.05. In all figures, significant differences are indicated by distinct italic letters; groups sharing the same letter are not significantly different.

### Reporting summary

Further information on research design is available in the Nature Portfolio Reporting Summary linked to this article.

## Supplementary information

Supplementary Information

Reporting Summary

## Data Availability

The source data supporting the findings from this study are available via the CUHK Research Data Repository at 10.48668/WDGXNE^53^. All parameters, structures, and MD simulation inputs/outputs for each system investigated in this study are publicly accessible through Figshare at 10.6084/m9.figshare.30950858^54^. The Uncropped (or less cropped with all lanes remained), unedited gel images are provided in Supplementary Fig. 21. The raw mass spec data and associated metadata are publicly accessible through Zenodo repository at 10.5281/zenodo.18487762^55^.
