# Supplementary material for: Calcium activation mechanism of a noncanonical aromatic L-amino acid decarboxylase from psilocybin mushroom Psilocybe cubensis
Source: Commun Biol. 2026 Feb 26;9:497. doi: 10.1038/s42003-026-09756-y (PMC13062060; doi:10.1038/s42003-026-09756-y)
Supplement: Supplementary file 1 — Supplementary Information [file 42003_2026_9756_MOESM1_ESM.pdf]

Supplementary Information for

Calcium activation mechanism of a noncanonical aromatic L-amino acid decarboxylase from psilocybin mushroom *Psilocybe cubensis*

Authors:

Tianjie Li[1†], Erin E. Reynolds[2,3,4,5†], Ziqi Wang[1], Michael P. Torrens-Spence[5], Jing-Ke Weng[2,4,5*], Yi Wang[1*]

Affiliations:

[1]Department of Physics, The Chinese University of Hong Kong, Shatin, New Territories, Hong Kong SAR, China

[2]Institute for Plant-Human Interface, Northeastern University, Boston, MA 02115, USA

[3]Department of Chemical Engineering, Massachusetts Institute of Technology, Cambridge, MA 02139, USA

[4]Department of Chemistry and Chemical Biology, Department of Bioengineering, and Department of Chemical Engineering, Northeastern University, Boston, MA 02115, USA

[5]Whitehead Institute for Biomedical Research, Cambridge, MA 02142, USA

[†]These authors contributed equally to this work.

*Correspondence: jingke.weng@northeastern.edu and yiwang@cuhk.edu.hk

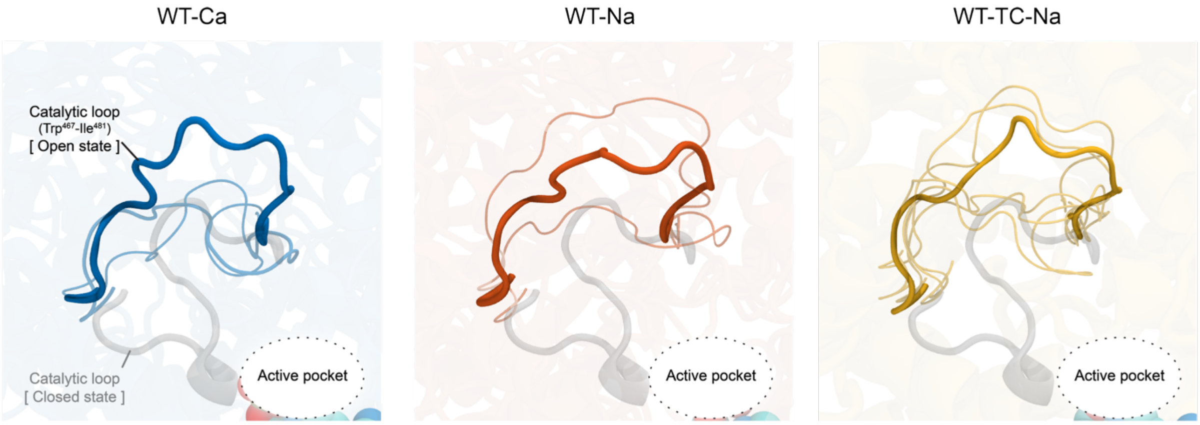

Fig. S1. Representative structures of the catalytic loop revealed by clustering analyses of WT-Ca, WT-Na and WT-TC-Na. The closed state conformation of the catalytic loop determined by the Holo-WT-Ca simulations (see Methods) is displayed in a transparent gray cartoon representation. The centroid structure of each cluster is depicted by cartoon representations whose thickness is scaled by relative population, with the top cluster shown as opaque and smaller clusters as transparent.

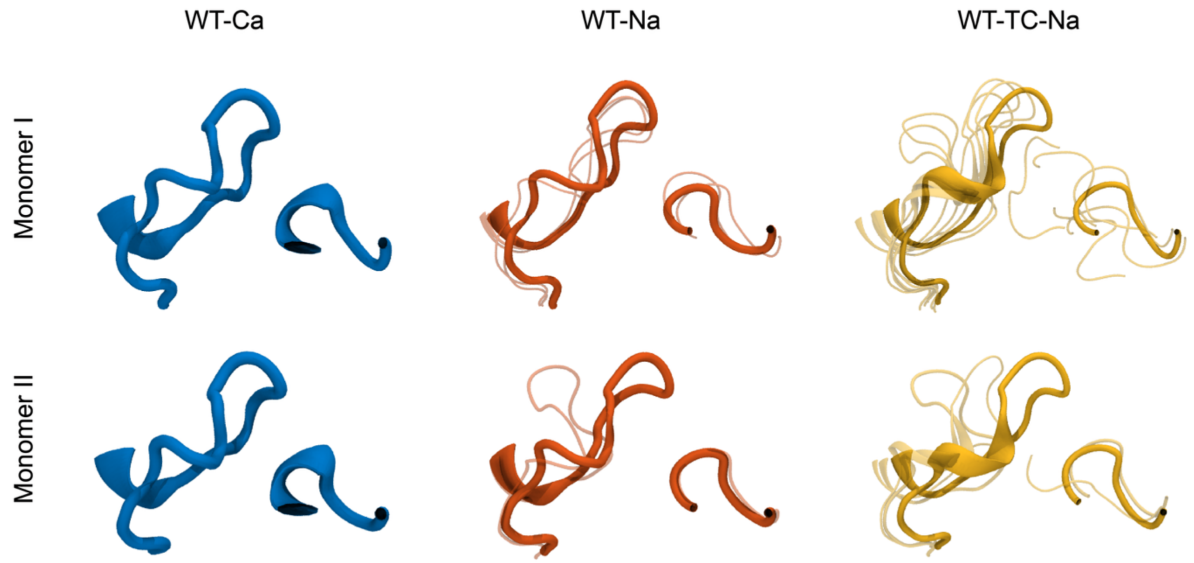

Fig. S2. Representative snapshots of the capping lid-rim structure of WT-Ca, WT-Na and WT-TC-Na revealed by clustering analyses on each monomer. The centroid structure of each cluster is depicted by cartoon representations whose thickness is scaled by relative population, with the top cluster shown as opaque and smaller clusters as transparent.

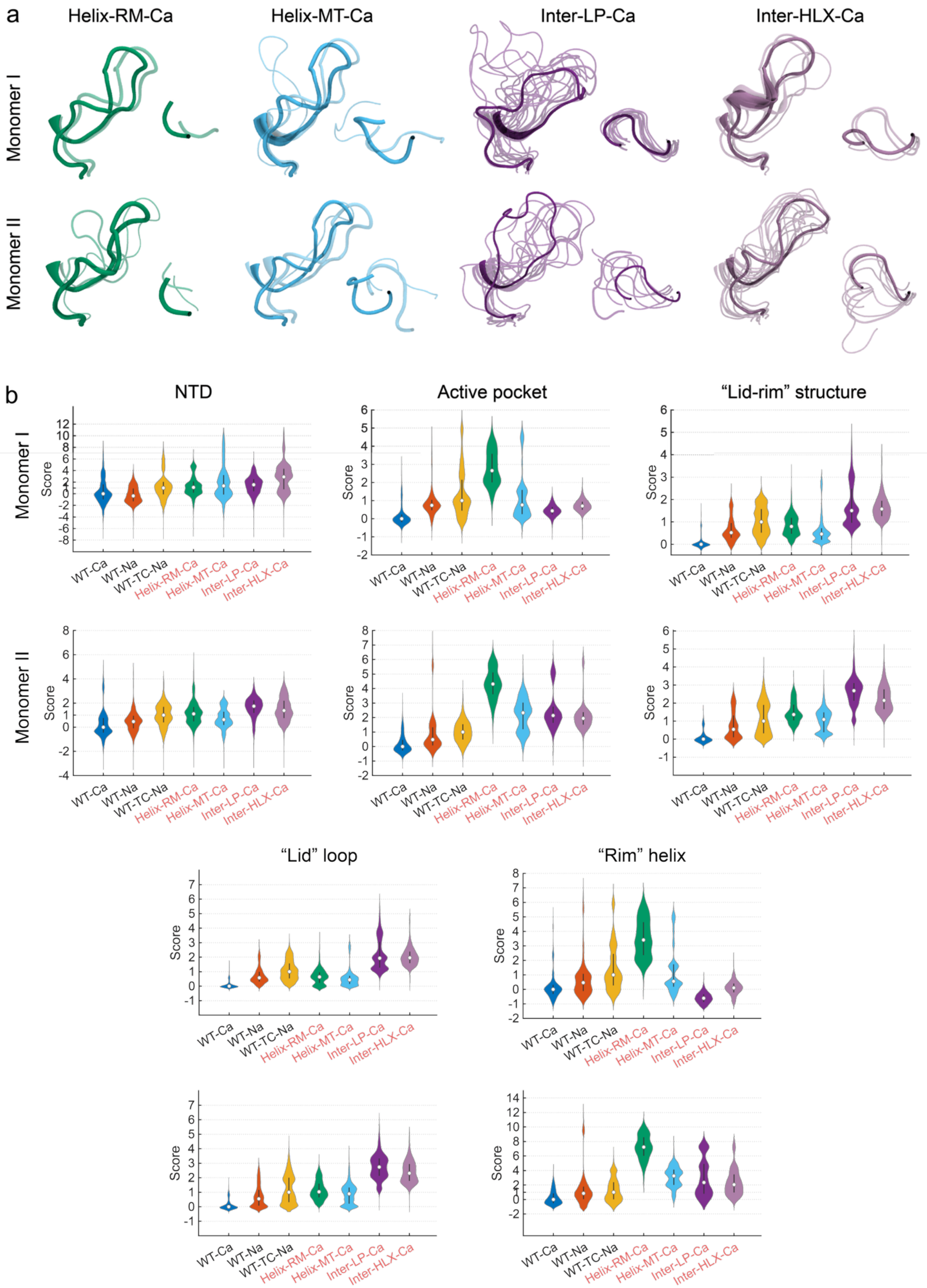

a
Helix-RM-Ca
Helix-MT-Ca
Inter-LP-Ca
Inter-HLX-Ca
Monomer I
Monomer II
b
NTD
Active pocket
"Lid-rim" structure
"Lid" loop
"Rim" helix
Score
WT-Ca
WT-Na
WT-TC-Na
Helix-RM-Ca
Helix-MT-Ca
Inter-LP-Ca
Inter-HLX-Ca

Fig. S3. Mutants with disruptive mutations on the capping lid-rim structure analyzed for each monomer. **a.** Representative snapshots of the capping lid-rim structure of the mutants with the rim helical structure destroyed (Helix-RM-Ca: green, Helix-MT-Ca: blue) or the lid-rim hydrophobic interface disrupted (Inter-LP-Ca: purple, Inter-HLX-Ca: pink) revealed by clustering analyses for each monomer. The centroid structure of each cluster is depicted by cartoon representations whose thickness is scaled by relative population, with the top cluster shown as opaque and smaller clusters as transparent. **b.** RCV scores. The median values and interquartile ranges are indicated by white markers and black bars, respectively.

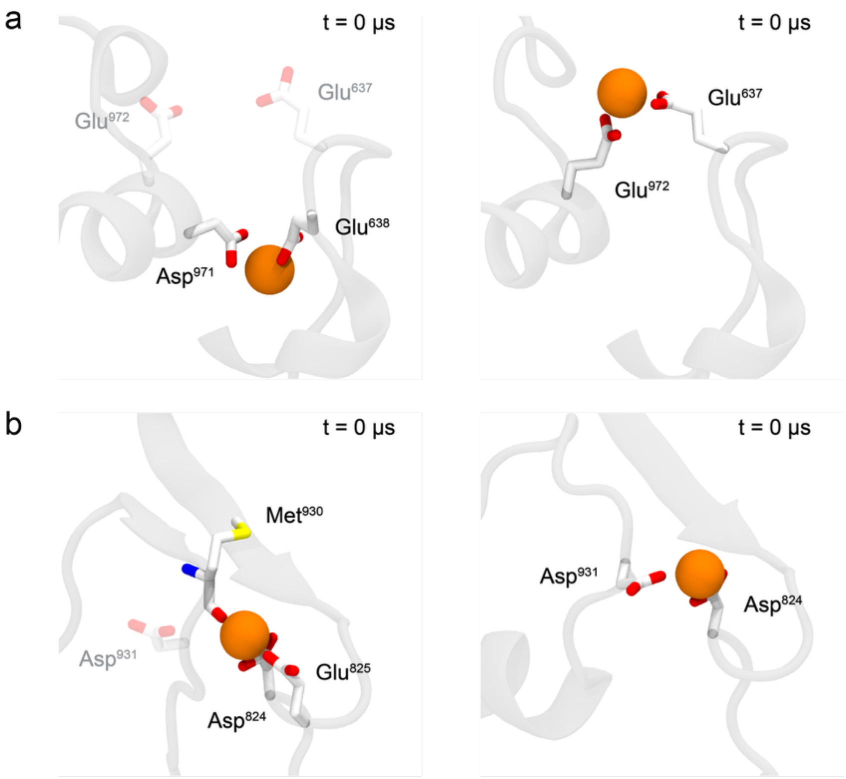

Fig. S4. Alternative binding of backup residues at the two metal-binding sites revealed in the MD simulations of WT-Ca.

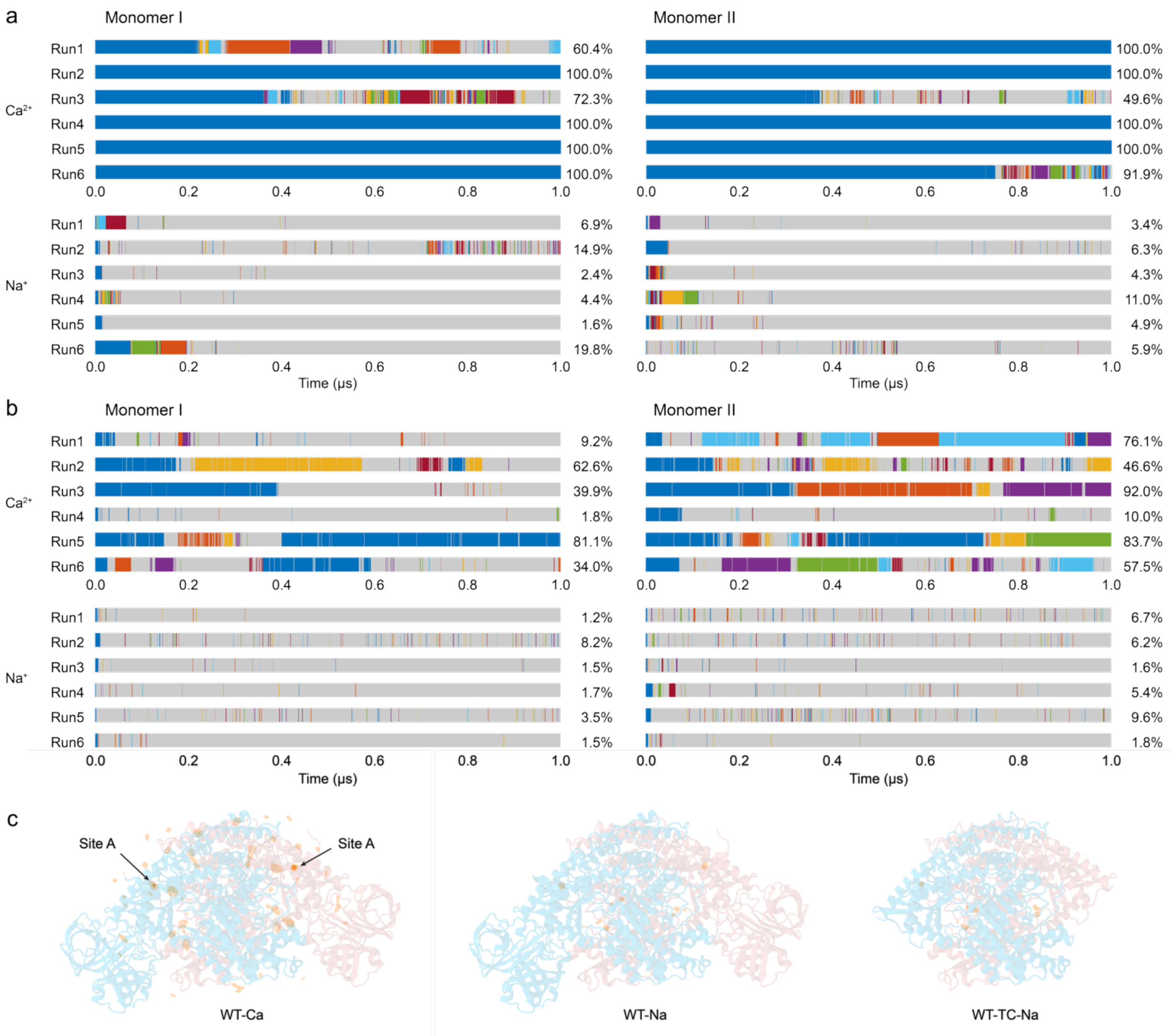

Fig. S5. Ion residence at metal-binding site in WT simulations. **a-b.** Residence of Ca^{2+} and Na^{+} at the interdomain site A between NTD and CTD (**a**), and the intradomain site B within the CTD (**b**). All the panels of each replica represent the residence of different ions with individual colors, whereas gray indicates no resident ion. **c.** Ion occupancy. Occupancy > 0.10 is shown in solid representation, whereas occupancy > 0.01 is shown transparently.

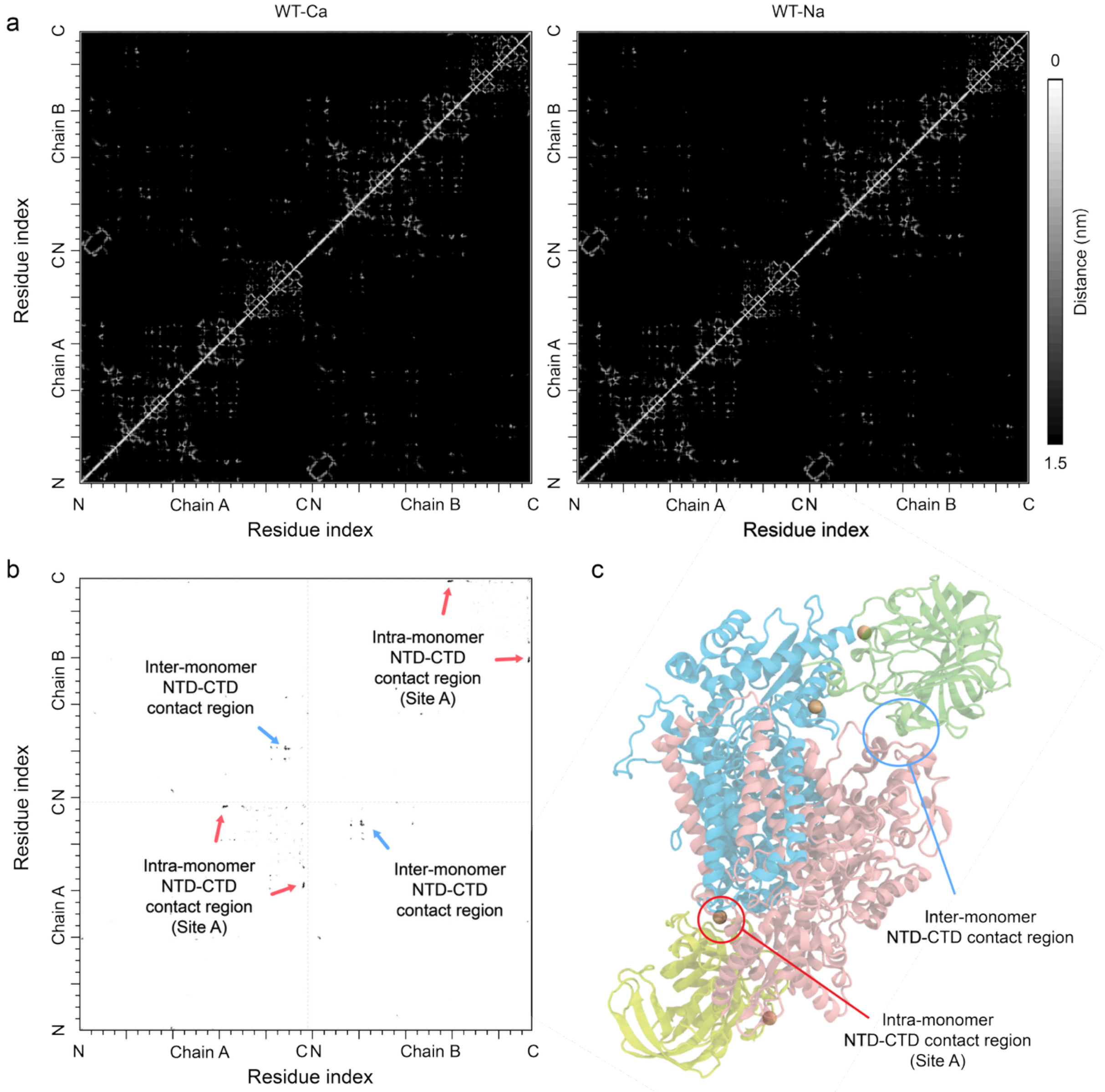

Fig. S6. Protein contact maps showing the disruption of site A and detach of CTD in WT-Na. **a.** Protein contact map of WT-Ca and WT-Na. **b.** Differential contact map of WT-Ca and WT-Na. Regions showing notable differences are indicated by arrows. **c.** The corresponding regions indicated by panel **b**.

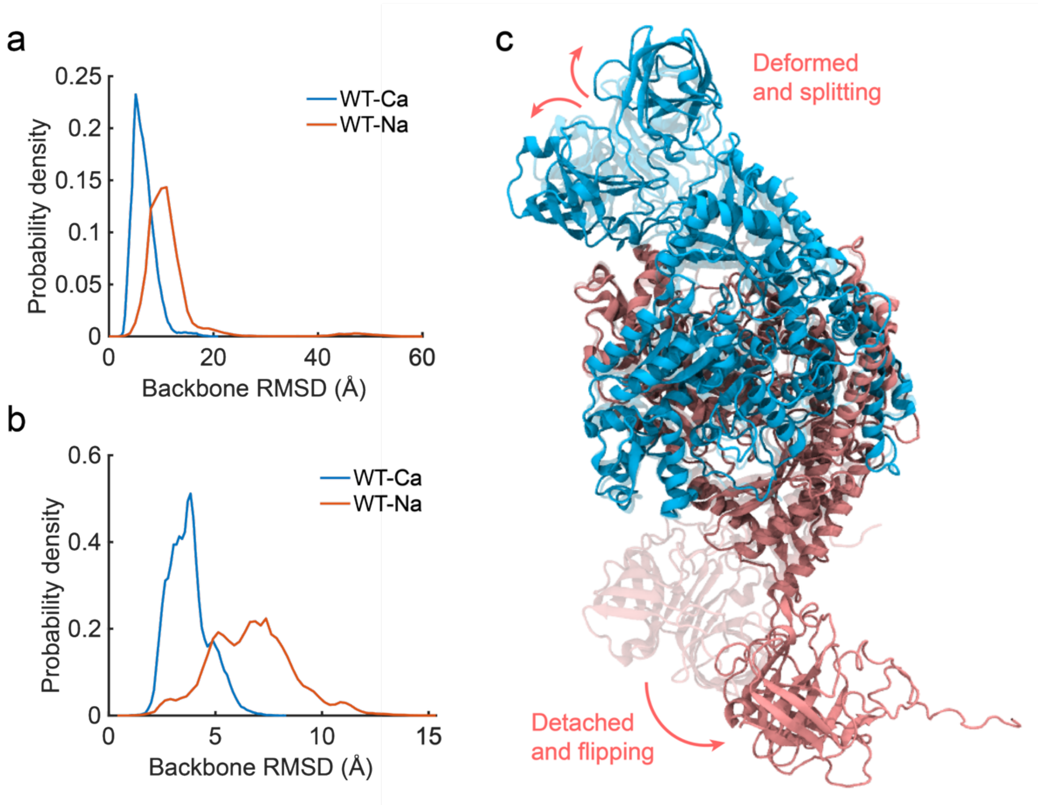

Fig. S7. Global and local conformational change of CTD in WT-Ca and WT-Na. **a.** Probability density of CTD global RMSD calculated from trajectories aligned by NTD. **b.** Probability density of CTD local RMSD calculated from trajectories aligned by the CTD itself. **c.** Snapshot showing the global and local disruption of CTD observed in WT-Na simulations.

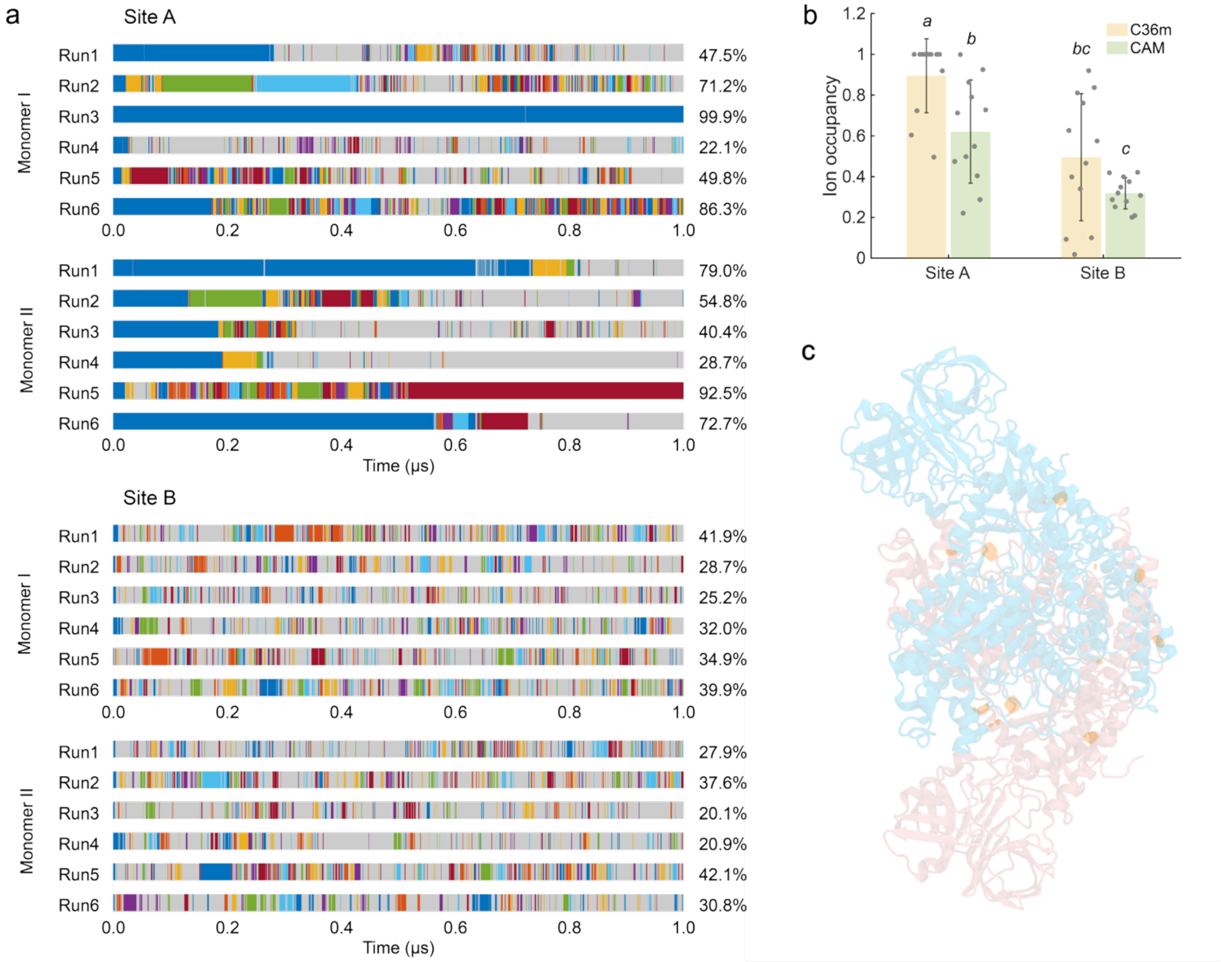

Fig. S8. Simulations of WT-Ca where the calcium ions are alternatively described by the multi-site model (CAM) showing lower occupancy of the Ca^{2+} at both metal-binding sites with a high exchange rate. **a.** Ca^{2+} residence. All the panels of each replica represent the residence of different ions with individual colors, whereas gray indicates no resident ion. **b.** Ion occupancy in CAM simulations in comparison with that in classic CHARMM simulations (C36m) (n=12; 2 monomers from 6 replicas). **c.** Snapshot showing ion occupancy. Occupancy > 0.10 is shown opaquely, whereas occupancy > 0.01 is shown transparently. The error bars represent the standard deviation of the replica means, whereas statistically significant differences between groups ($p < 0.05$) are indicated by italic letters.

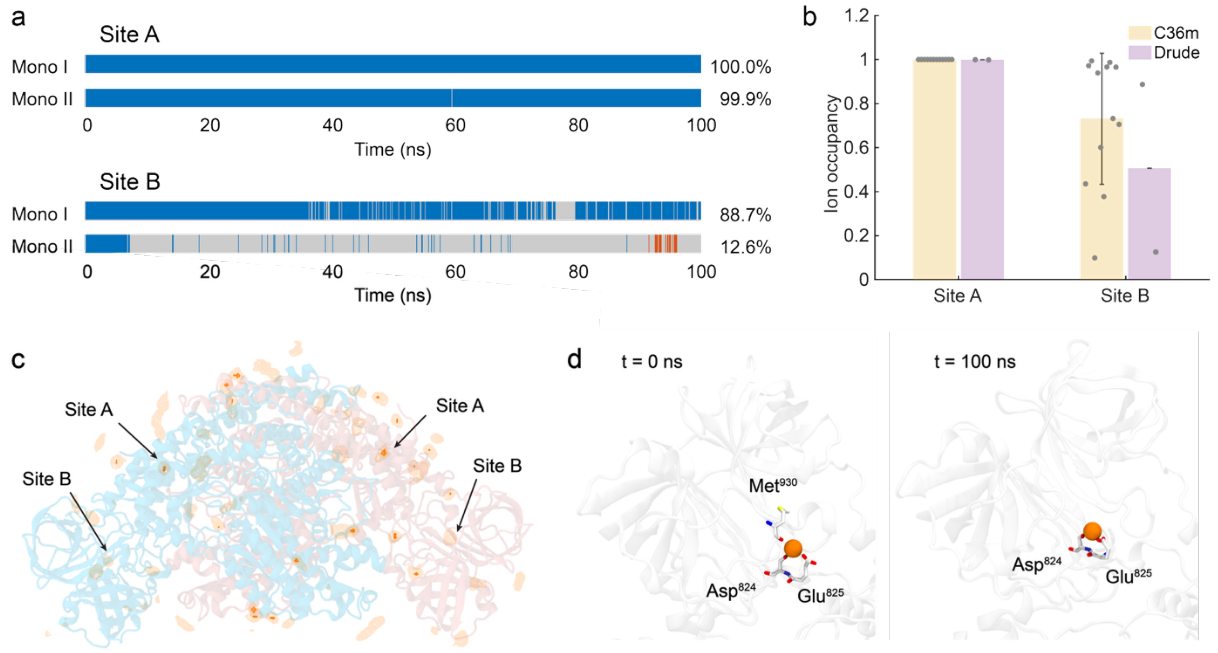

Fig. S9. A 100-ns Drude polarizable simulation of the full-length *Pc*ncAAAD revealing the ion residence at both metal-binding sites. **a.** Ca^{2+} residence at site A and B in each monomer (Mono). All the panels of each replica represent the residence of different ions with individual colors, whereas gray indicates no resident ion. **b.** Ion occupancy in the Drude simulations compared to the first 100-ns period of classic CHARMM simulations of WT-Ca (C36m) (n=12 for C36m; 2 monomers from 6 replicas). The error bars represent the standard deviation of the replica means. **c.** Ca^{2+} occupancy of the Drude simulations. Occupancy > 0.10 is shown opaquely, whereas occupancy > 0.01 is shown transparently. **d.** Snapshots showing the nearly stationary Ca^{2+} ions at site B of monomer II even after unbinding from the other β-barrel repeat.

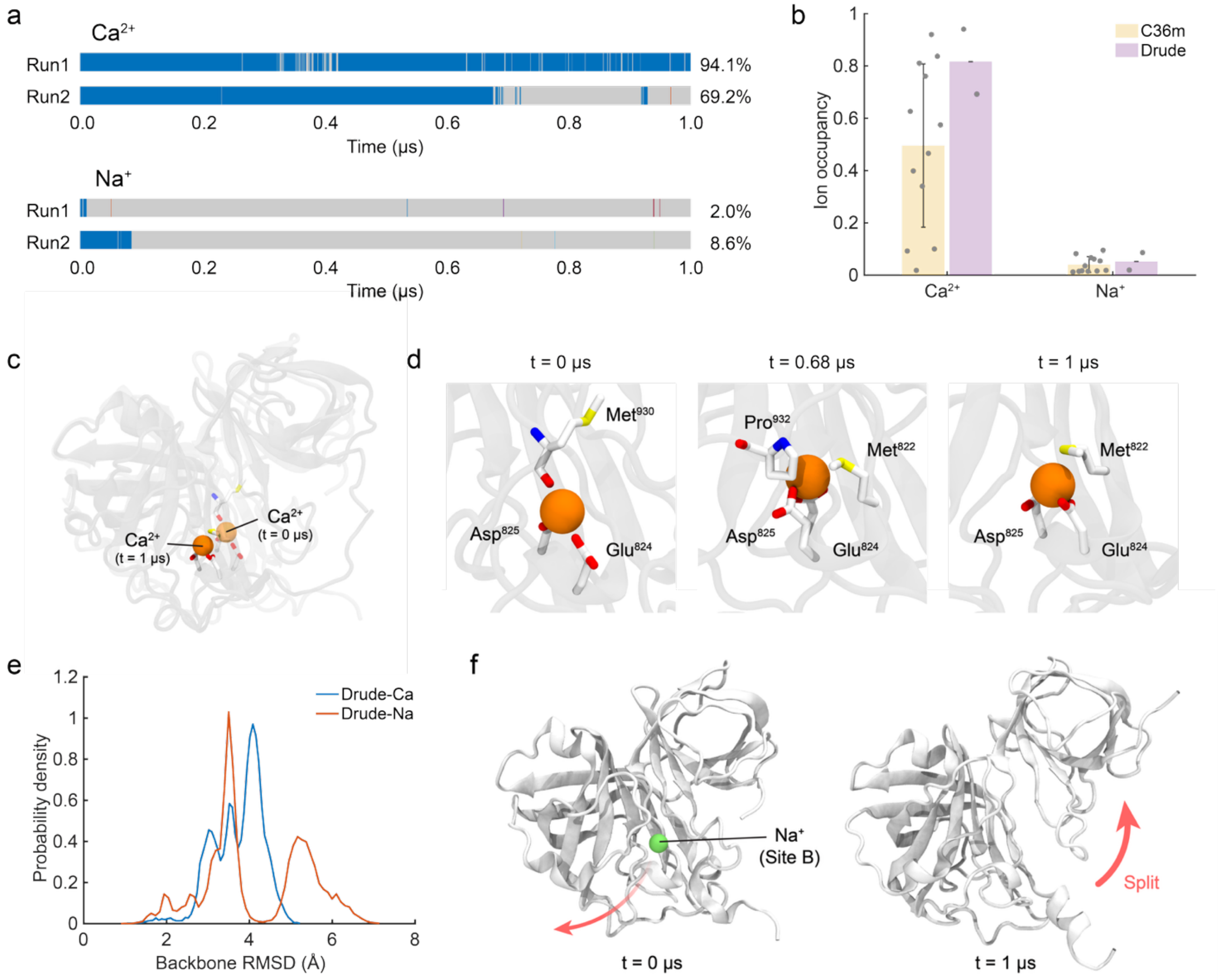

Fig. S10. Drude polarizable simulations of the CTD segment revealing a higher Ca^{2+} ion residence at site B than classic CHARMM simulations (C36m). **a.** Ca^{2+} and Na^{+} residence at site B. All the panels of each replica represent the residence of different ions with individual colors, whereas gray indicates no resident ion. **b.** Ion occupancy at site B in Drude simulations compared to that in the C36m simulations of full-length dimer (n=12 for C36m; 2 monomers from 6 replicas). The error bars represent the standard deviation of the replica means. **c.** Ca^{2+} unbinding event at site B captured in run2 of the Drude simulations. **d.** Snapshots showing the process of the Ca^{2+} unbinding event at site B. **e.** Probability density of CTD's local RMSD. **f.** Snapshots showing the split of the two β-barrels in simulations with sodium.

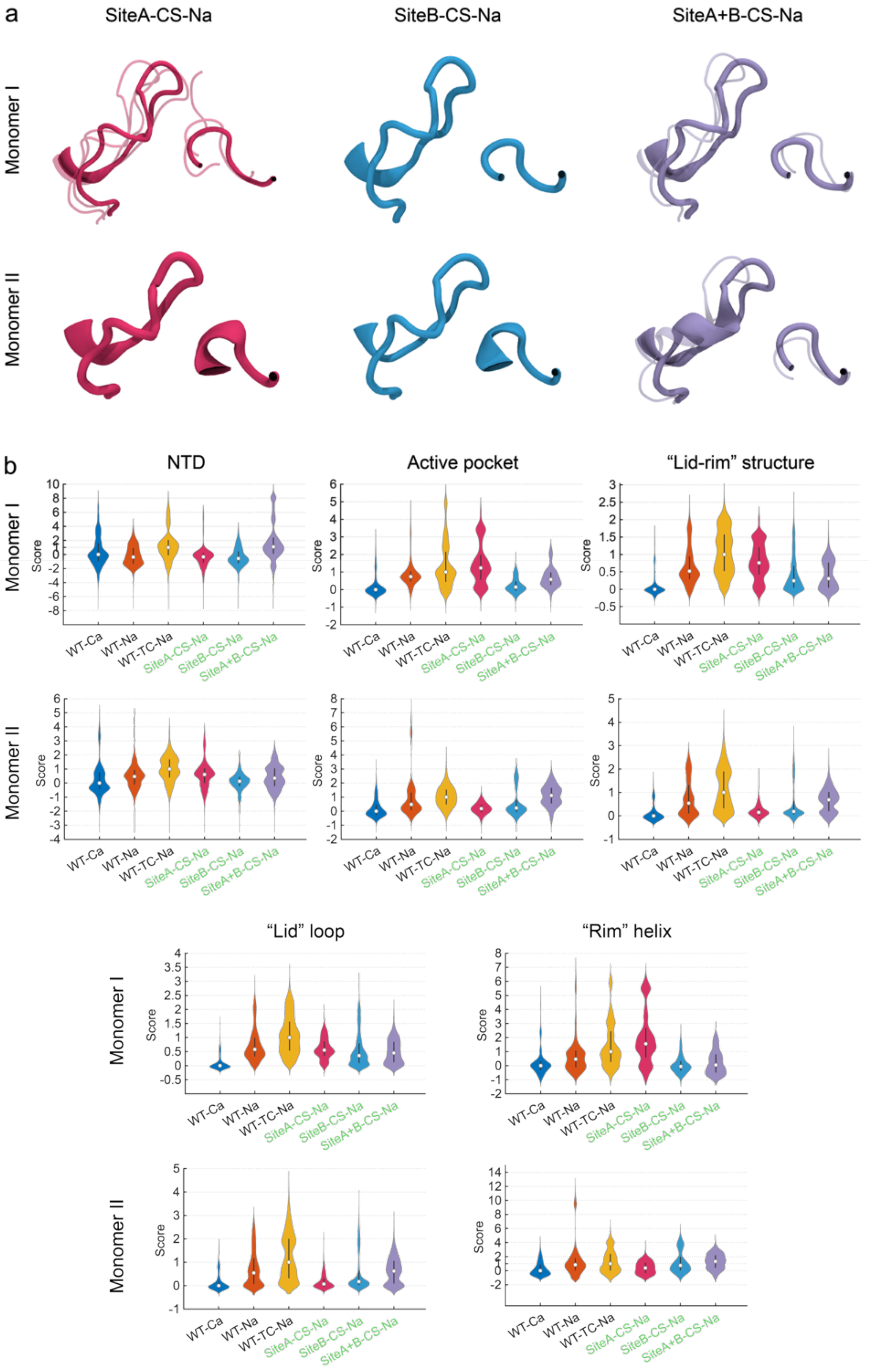
a
SiteA-CS-Na
SiteB-CS-Na
SiteA+B-CS-Na
Monomer I
Monomer II
b
NTD
Active pocket
"Lid-rim" structure
"Lid" loop
"Rim" helix
Score
WT-Ca
WT-Na
WT-TC-Na
SiteA-CS-Na
SiteB-CS-Na
SiteA+B-CS-Na

Fig. S11. Mutants with disruptive mutations on the metal-binding sites analyzed for each monomer. **a.** Representative snapshots of the capping lid-rim structure of the mutants with disrupted site A (SiteA-MT-Ca), site B (SiteB-MT-Ca), or both sites (SiteA+B-MT-Ca) revealed by clustering analyses for each monomer. The centroid structure of each cluster is depicted by cartoon representations whose thickness is scaled by relative population, with the top cluster shown as opaque and smaller clusters as transparent. **b.** RCV scores. The median values and interquartile ranges are indicated by white markers and black bars, respectively.

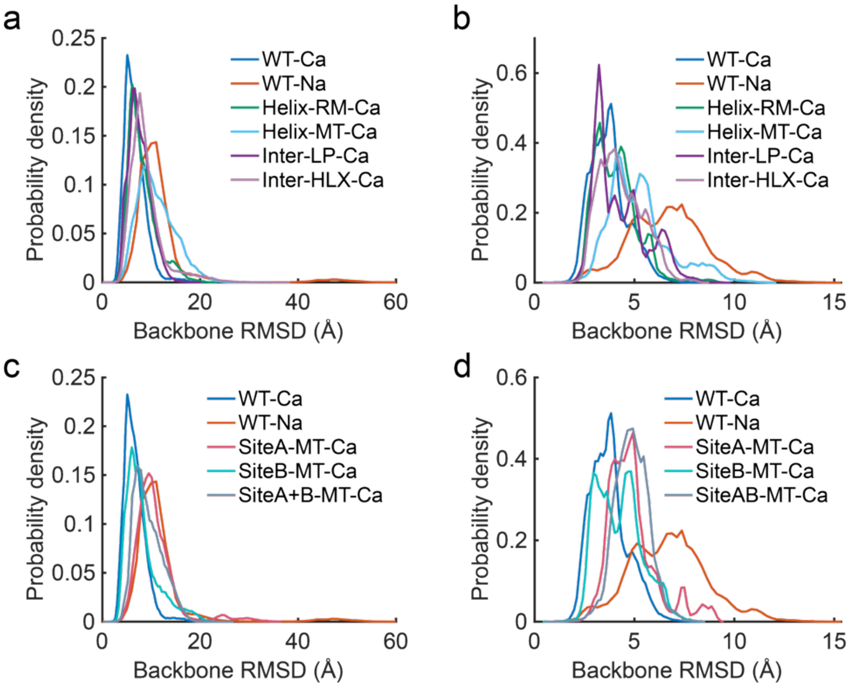

Fig. S12. Global and local CTD conformational change of the mutants with respect to the trajectories aligned by the dimeric NTD and the CTD itself, respectively. **a, b.** Global (**a**) and local (**b**) CTD conformational change of the lid-rim structure mutants. **c, d.** Global (**c**) and local (**d**) CTD conformational change of the metal-binding site mutants.

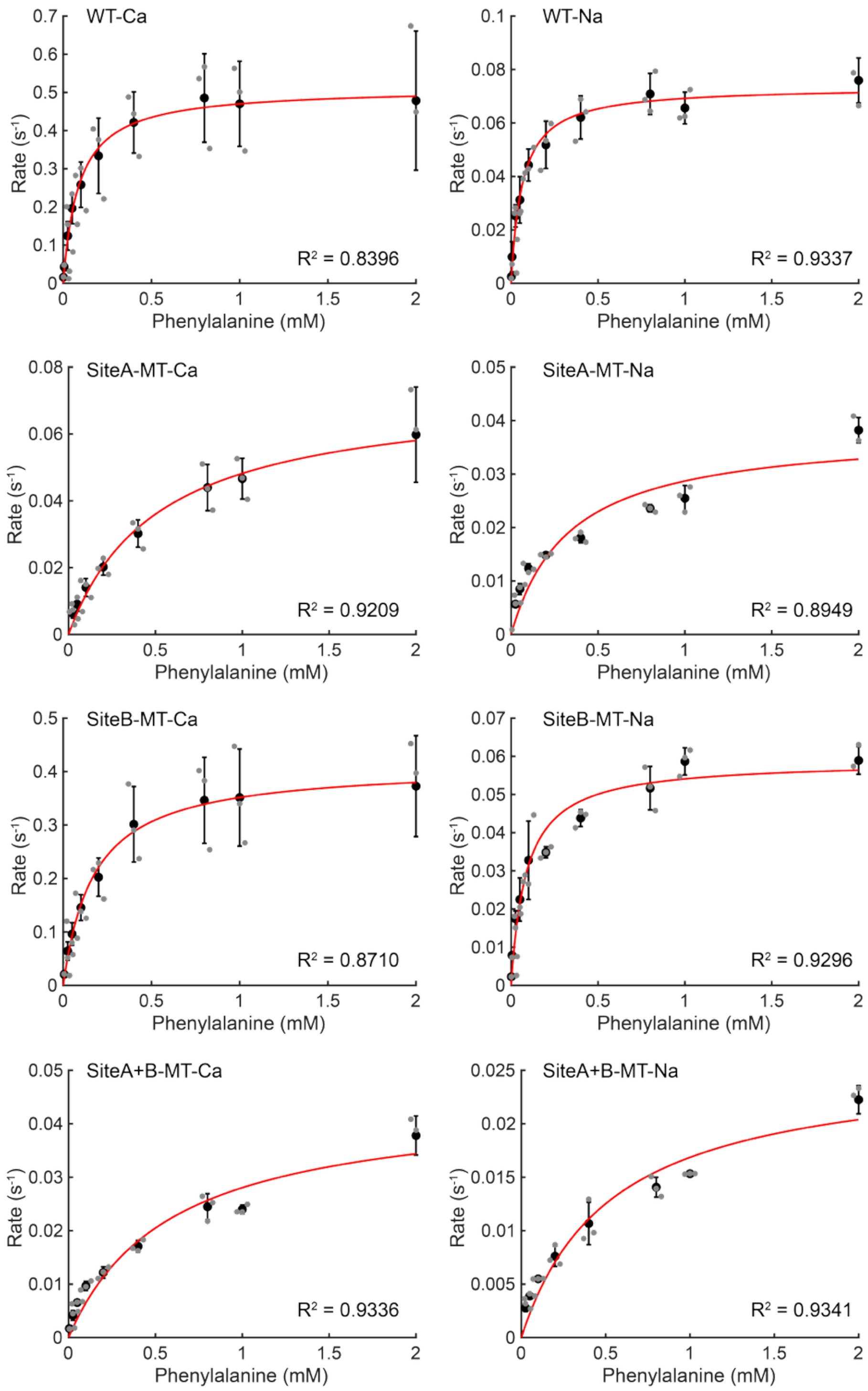

Fig. S13. Kinetic data for wildtype *Pc*ncAAAD and mutants measured at concentrations of L-Phenylalanine from 1 µM to 2 mM (n=3). Measurements performed in triplicate. Non-linear regression performed using the Michaelis-Menten model in GraphPad Prism v. 8.4.3.

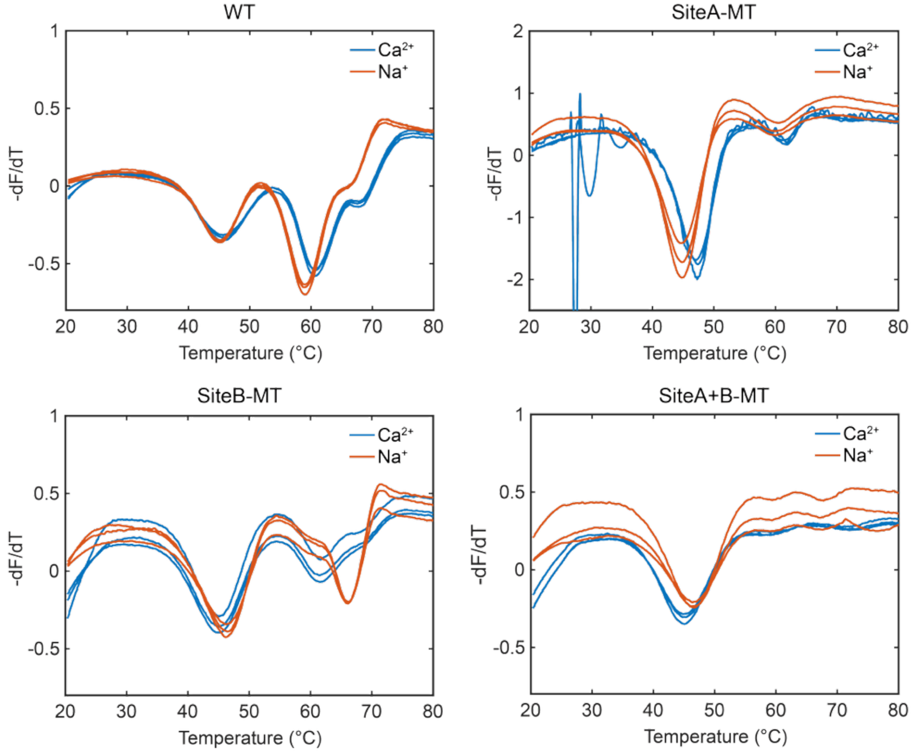

Fig. S14. ThermoFluor assay melting curves indicate that mutations at calcium-binding sites A and B decrease stability of *Pc*ncAAAD. Melting curves were gathered for wild-type *Pc*ncAAAD (WT), SiteA-MT, SiteB-MT, and SiteA+B-MT mutants in both calcium (Ca^{2+}) and sodium (Na^{+}) buffer. Experiments performed in triplicate and the negative derivative of fluorescence over temperature is plotted (-dF/dT).

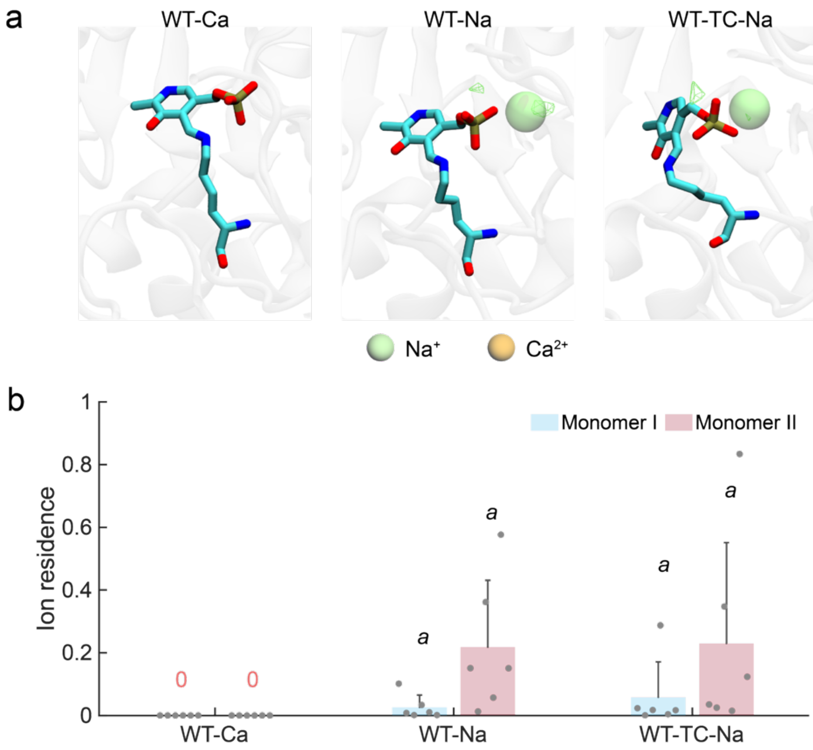

Fig. S15. Ion intrusion to the active pocket in the WT simulations. **a.** Ion occupancy nearby the internal aldimine (LLP). Occupancy of Ca^{2+} or Na^{+} larger than 0.01 is indicated by mesh representations. **b.** Ion occupancy in the active pocket of each monomer (n=6). The error bars represent the standard deviation of the replica means, whereas statistically significant differences between non-zero groups ($p < 0.05$) are indicated by italic letters (see Method).

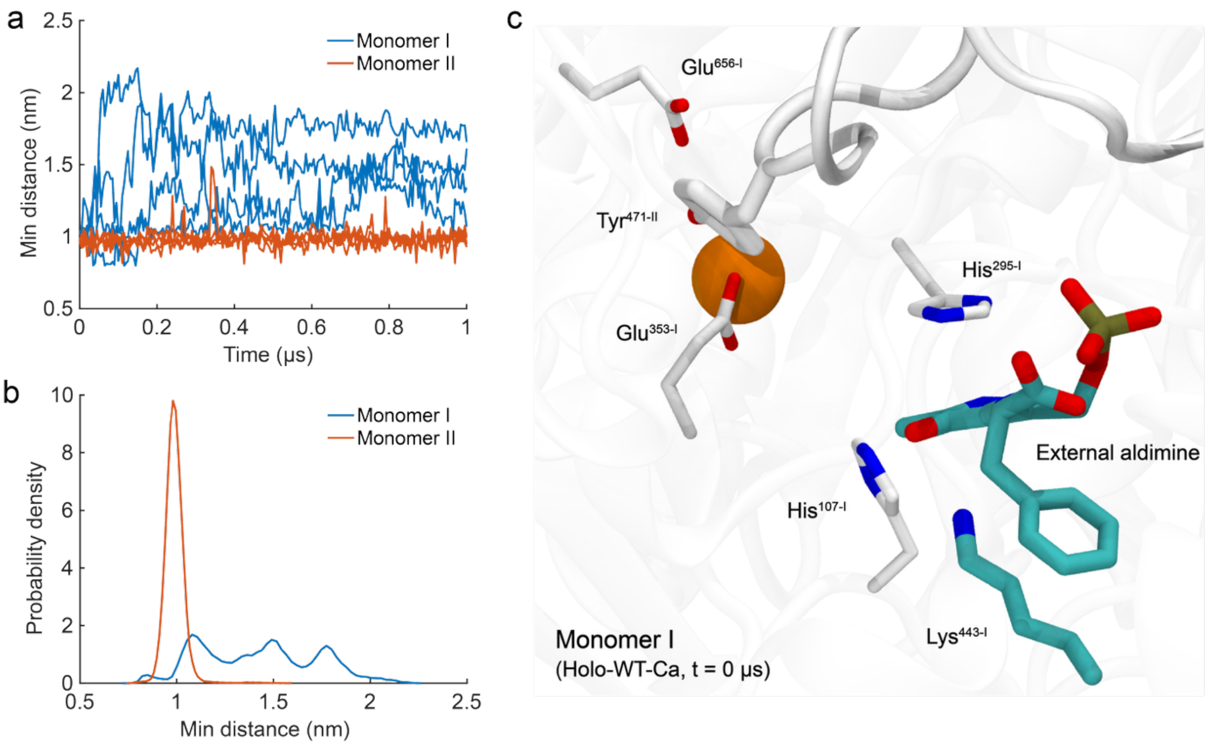

Fig. S16. Closed state of the catalytic loop featured by the minimum distance between Tyr471 and external aldimine in the holo simulations. **a.** Minimum distance between Tyr471 and external aldimine for each monomer during each replica. **b.** Probability density of the calculated minimum distance. **c.** Close-up of the active pocket of monomer I in the initial structure.

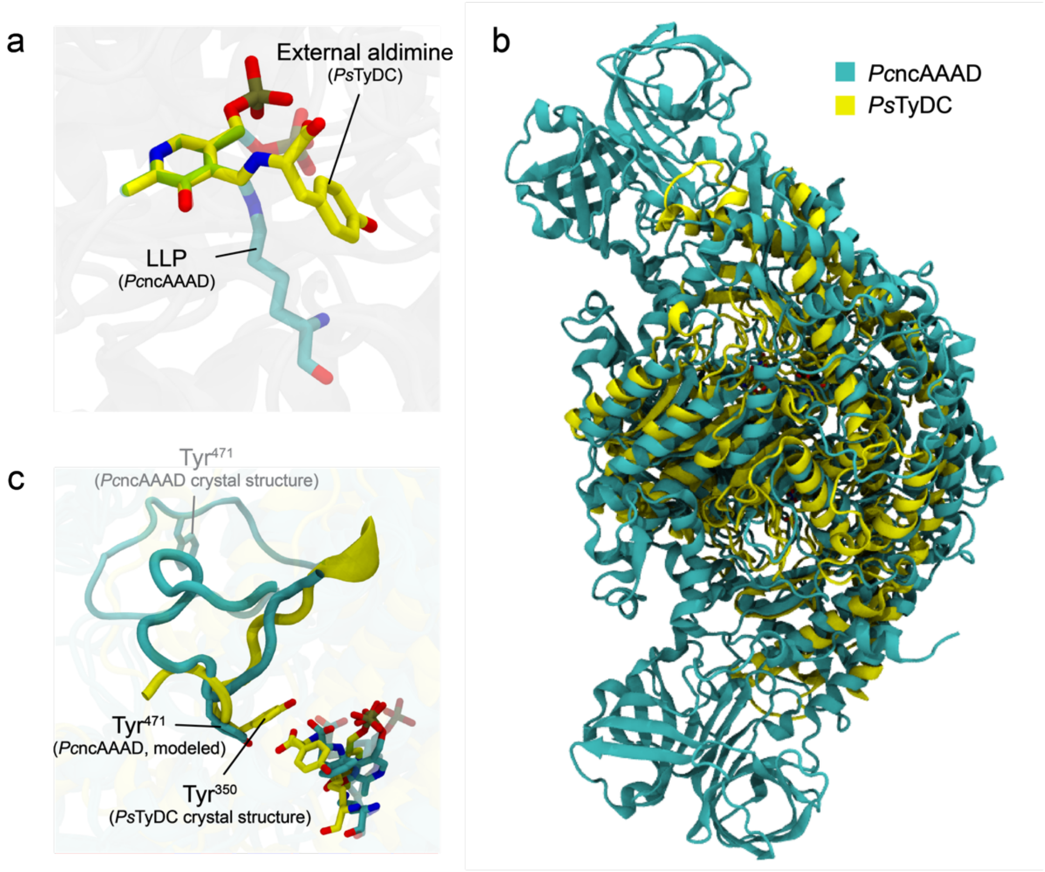

Fig. S17. Modeling of holo-form WT-Ca simulations with external aldimine through a two-step superposition method referring to the crystal structure of holo-form *Ps*TyDC (pdb: 6EEM). **a.** Superimposed structure of the external aldimine and LLP by the pyridine ring in the second step. **b.** The superimposition of *Pc*ncAAAD to *Ps*TyDC by the PLP core region through MultiSeq in the first step. **c.** The corresponding position of the catalytic tyrosine in the superimposition.

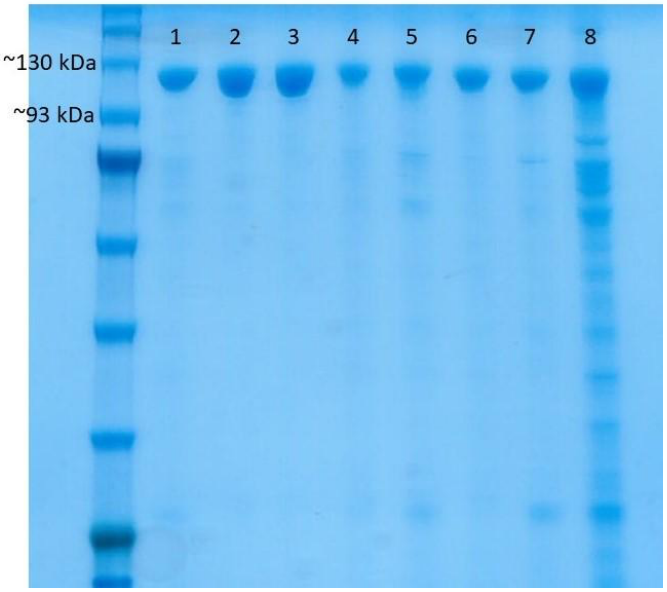

Fig. S18. SDS-PAGE gel used to estimate concentration of *Pc*ncAAAD wildtype and variants in activity assay. Gel was stained with coomassie brilliant blue G-250 dye. 1: WT, 2: Helix-RM, 3: Helix-MT, 4: Inter-HLX, 5: Inter-LP, 6: SiteB-MT, 7: SiteA-MT, 8: SiteA+B-MT. Image analyzed in ImageJ. Expected molecular weight of *Pc*ncAAAD is 113.2 kDa.

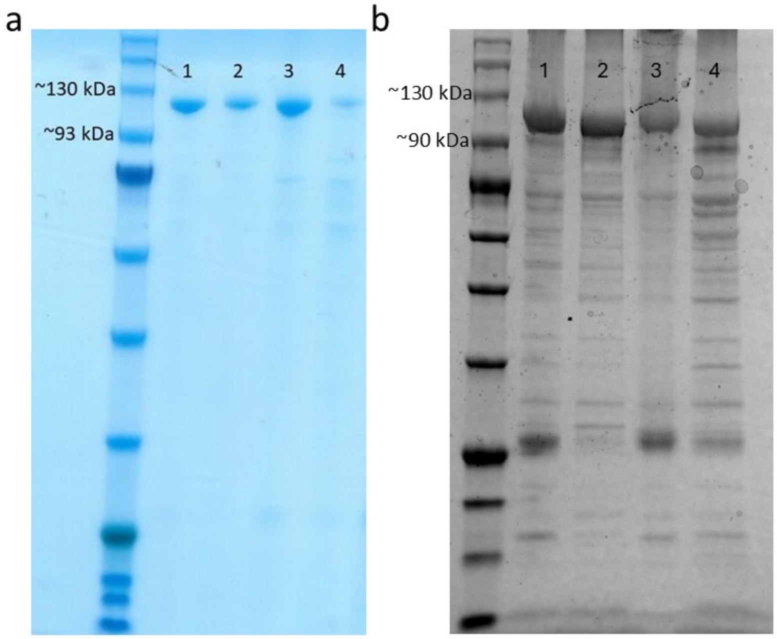

Fig. S19. a. SDS-PAGE gel used to estimate concentration of *Pc*ncAAAD wildtype and variants in enzyme kinetics reactions. 1: WT, 2: SiteB-MT, 3: SiteA-MT, 4: SiteA+B-MT. Image analyzed in ImageJ. Expected molecular weight of *Pc*ncAAAD is 113.2 kDa. **b**. An additional SDS-PAGE gel of *Pc*ncAAAD wildtype and variants to more clearly show the band in lane 4. 1: WT, 2: SiteB-MT, 3: SiteA-MT, 4: SiteA+B-MT. Expected molecular weight of *Pc*ncAAAD is 113.2 kDa.

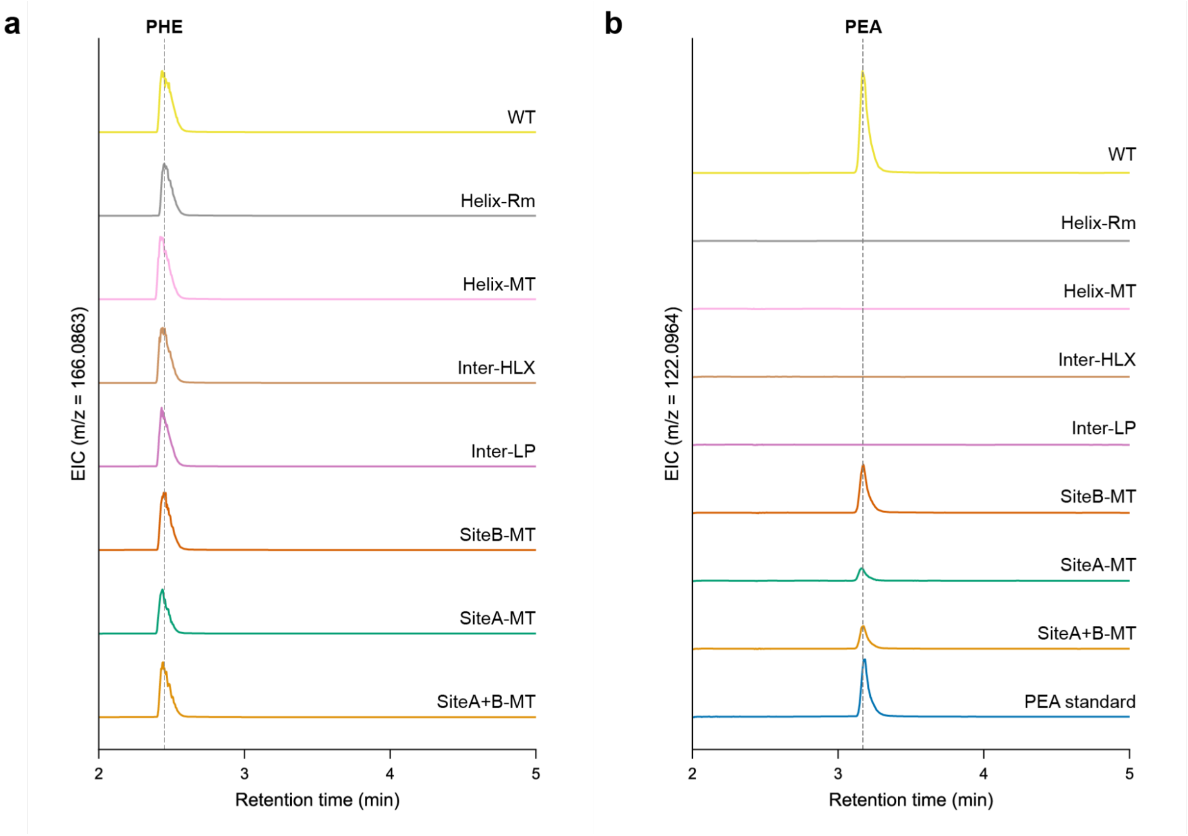

Fig. S20. a. Representative liquid chromatography mass spectrometry (LC-MS) traces out of n=3 replicates for *Pc*ncAAAD enzyme assays performed with wildtype (WT) and mutants. Extracted ion chromatogram (EIC) is shown for substrate phenylalanine (PHE) $[M+H]^+$ ion (*m/z* =166.0863). **b**. Representative LC-MS traces out of n=3 replicates for *Pc*ncAAAD enzyme assays performed with wildtype (WT) and mutants. EIC is shown for product phenylethylamine (PEA) $[M+H]^+$ ion (*m/z* =122.0964).

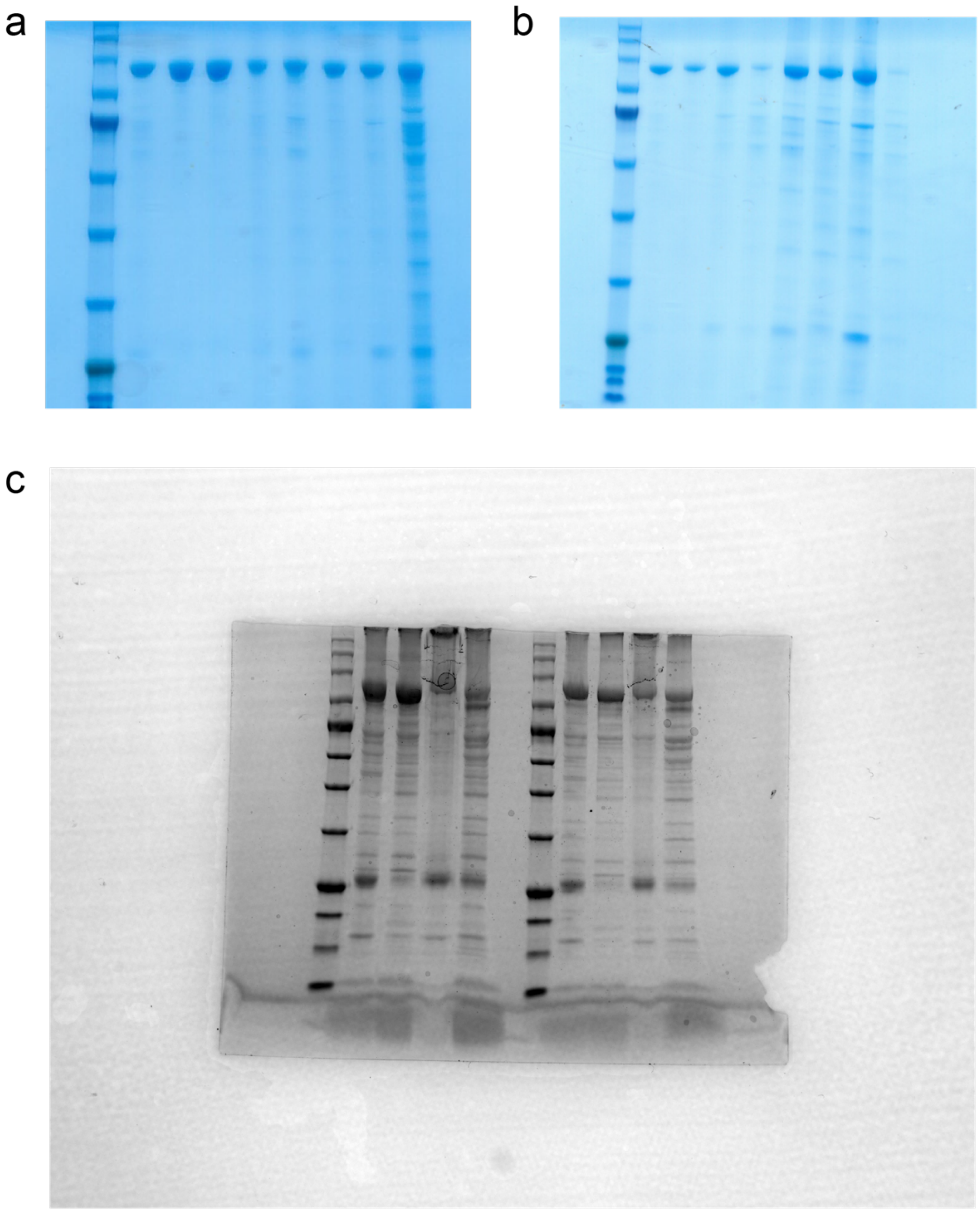

Fig. S21. Uncropped (or less cropped with all lanes remained), unedited gel images for Fig S18 (a) and Fig S19 (b-c).

Table S1. pKa values of His442 and LLP443 calculated using PROPKA 3[1,2].

Group	pKa	Model pKa
His442-I-imidazole	9.70	6.50
LLP443-I-phosphate-OP2	4.94	6.00
LLP443-I-phosphate-OP3	7.26	6.00
His442-II-imidazole	9.81	6.50
LLP443-II-phosphate-OP2	4.69	6.00
LLP443-II-phosphate-OP3	6.99	6.00

Table S2. RCV score determined by median. The likelihood of deactivation is indicated by the score level: ▲ denotes a score between wt-na and 1.00; ▲▲ denotes a score exceeding 1.00.

	NTD	Active pocket	Lid-rim	Lid loop	Rim helix
WT-Ca	0.00	0.00	0.00	0.00	0.00
WT-Na	0.11▲	0.84▲	0.54▲	0.56▲	0.50▲
WT-TC-Na	1.00▲▲	1.00▲▲	1.00▲▲	1.00▲▲	1.00▲▲
Helix-RM-Ca	1.11▲▲	3.66▲▲	1.03▲▲	0.78▲	4.93▲▲
Helix-MT-Ca	0.76▲	1.61▲▲	0.65▲	0.57▲	1.66▲▲
Inter-LP-Ca	1.73▲▲	1.11▲▲	1.98▲▲	2.21▲▲	-0.05
Inter-HLX-Ca	1.97▲▲	1.27▲▲	1.78▲▲	2.06▲▲	0.65▲
SiteA-MT-Ca	0.54▲	0.40	0.75▲	0.92▲	-0.06
SiteB-MT-Ca	-0.24	0.24	0.19	0.29	-0.34
SiteA+B-MT-Ca	0.58▲▲	0.80	0.93▲	1.04▲▲	-0.35

Table S3. Average T_m values for wild-type *Pc*ncAAAD (WT) and mutants calculated from thermofluor assay melting curves. Errors represent the standard deviations.

	T_m sodium (°C)	T_m calcium (°C)	Rightward shift (°C)
WT	59.5 +/- 0.1	61.2 +/- 0.1	+1.8
SiteB-MT	46.6 +/- 0.1	45.1 +/- 0.1	-1.5
SiteA-MT	45.1 +/- 0.0	47.3 +/- 0.2	+2.3
SiteA+B-MT	46.7 +/- 0.1	45.0 +/- 0.2	-1.7

Reference

1. Søndergaard, C. R., Olsson, M. H. M., Rostkowski, M. & Jensen, J. H. Improved treatment of ligands and coupling effects in empirical calculation and rationalization of pKa values. *J. Chem. Theory Comput.* **7**, 2284–2295 (2011).
2. Olsson, M. H. M., Søndergaard, C. R., Rostkowski, M. & Jensen, J. H. PROPKA3: Consistent treatment of internal and surface residues in empirical pKa predictions. *J. Chem. Theory Comput.* **7**, 525–537 (2011).
