# Supplementary material for: Calcium activation mechanism of a noncanonical aromatic L-amino acid decarboxylase from psilocybin mushroom Psilocybe cubensis
Source: Commun Biol. 2026 Feb 26;9:497. doi: 10.1038/s42003-026-09756-y (PMC13062060; doi:10.1038/s42003-026-09756-y)
Supplement: Supplementary file 2 — Reporting Summary [file 42003_2026_9756_MOESM2_ESM.pdf]

Corresponding author(s): Yi Wang
Last updated by author(s): Feb 8, 2026

Reporting Summary

Nature Portfolio wishes to improve the reproducibility of the work that we publish. This form provides structure for consistency and transparency in reporting. For further information on Nature Portfolio policies, see our Editorial Policies and the Editorial Policy Checklist.

Statistics

For all statistical analyses, confirm that the following items are present in the figure legend, table legend, main text, or Methods section.

n/a	Confirmed	
☐	☒	The exact sample size (*n*) for each experimental group/condition, given as a discrete number and unit of measurement
☐	☒	A statement on whether measurements were taken from distinct samples or whether the same sample was measured repeatedly
☐	☒	The statistical test(s) used AND whether they are one- or two-sided<br>*Only common tests should be described solely by name; describe more complex techniques in the Methods section.*
☐	☒	A description of all covariates tested
☐	☒	A description of any assumptions or corrections, such as tests of normality and adjustment for multiple comparisons
☐	☒	A full description of the statistical parameters including central tendency (e.g. means) or other basic estimates (e.g. regression coefficient) AND variation (e.g. standard deviation) or associated estimates of uncertainty (e.g. confidence intervals)
☐	☒	For null hypothesis testing, the test statistic (e.g. *F*, *t*, *r*) with confidence intervals, effect sizes, degrees of freedom and *P* value noted<br>*Give P values as exact values whenever suitable.*
☒	☐	For Bayesian analysis, information on the choice of priors and Markov chain Monte Carlo settings
☒	☐	For hierarchical and complex designs, identification of the appropriate level for tests and full reporting of outcomes
☒	☐	Estimates of effect sizes (e.g. Cohen's *d*, Pearson's *r*), indicating how they were calculated

Our web collection on statistics for biologists contains articles on many of the points above.

Software and code

Policy information about availability of computer code

Data collection	Classical molecular dynamics simulations were conducted by GROMACS (version 2024.5) with CHARMM36 (version July 2022 update), CGenFF (version 4.6). Polarizable molecular dynamics simulations were modeled by Solution Builder and Drude Prepper on CHARMM-GUI web interface and conducted by NAMD (version 3.0.2) with Drude force field (version 2023 update). Modeling of the mutants was conducted by Modeller (version 10.7). The structural alignment was conducted by MultiSeq (version 3.1) in VMD (version 2.0.0a6). The protonation states of protein residues were predicted by PROPKA (version 3.5.1). Liquid-chromatography mass-spectrometry (LC-MS) peak areas were quantified using Thermo Scientific Freestyle (v. 1.8 SP). Protein melting points were calculated by LightCycler 480 Software (version release 1.5.1.62 SP2). Image J (1.53e) with Java (1.8.0_172) was used to calculate densitometry and estimate protein concentrations.
Data analysis	Molecular dynamics simulations were analyzed using GROMACS (version 2024.5), VMD (version 2.0.0a6), and MATLAB (version R2024b). Kinetic constants were determined by fitting in Prism (version 8.4.3). All statistical tests and analyses were conducted using Prism (version 10.4.1).

For manuscripts utilizing custom algorithms or software that are central to the research but not yet described in published literature, software must be made available to editors and reviewers. We strongly encourage code deposition in a community repository (e.g. GitHub). See the Nature Portfolio guidelines for submitting code & software for further information.

Data

Policy information about availability of data

All manuscripts must include a data availability statement. This statement should provide the following information, where applicable:

- Accession codes, unique identifiers, or web links for publicly available datasets
- A description of any restrictions on data availability
- For clinical datasets or third party data, please ensure that the statement adheres to our policy

The data supporting the findings from this study can be found within the paper, supplementary materials. The source data supporting the findings from this study is available via the Chinese University of Hong Kong (CUHK) Research Data Repository at https://doi.org/10.48668/WDGXNE. All parameters, structures, and MD simulation inputs/outputs for each system investigated in this study are publicly accessible through Figshare at https://doi.org/10.6084/m9.figshare.30950858. The uncropped (or less cropped with all lanes remained), unedited gel images are provided in Supplementary Fig. 21. The raw mass spec data and associated metadata are publicly accessible through Zenodo repository at https://doi.org/10.5281/zenodo.18487762.

Research involving human participants, their data, or biological material

Policy information about studies with human participants or human data. See also policy information about sex, gender (identity/presentation), and sexual orientation and race, ethnicity and racism.

Reporting on sex and gender	N/A
Reporting on race, ethnicity, or other socially relevant groupings	N/A
Population characteristics	N/A
Recruitment	N/A
Ethics oversight	N/A

Note that full information on the approval of the study protocol must also be provided in the manuscript.

Field-specific reporting

Please select the one below that is the best fit for your research. If you are not sure, read the appropriate sections before making your selection.

☒ Life sciences ☐ Behavioural & social sciences ☐ Ecological, evolutionary & environmental sciences

For a reference copy of the document with all sections, see nature.com/documents/nr-reporting-summary-flat.pdf

Life sciences study design

All studies must disclose on these points even when the disclosure is negative.

Sample size	No statistical methods were used to predetermine sample size. Sample size and simulation time length was based on preliminary simulations and experimentation. The sample size is sufficient to identify differences between groups. Details regarding sample size of all simulations and experiments are provided in the Methods section and figure legends.
Data exclusions	No data were excluded from the analyses.
Replication	All classical molecular dynamics simulations were performed in at least four replicas, whereas polarizable simulations were performed in two replicas (for C-terminal segment) and one replica (for full-length protein). All experiments were performed at least three times. The results were reliably reproducible.
Randomization	All random seeds used in simulations were automatically generated. All samples and cells were allocated randomly into experimental groups.
Blinding	In all simulations and experiments, investigators remained blind to the group allocation during data collection and analyses.

Reporting for specific materials, systems and methods

We require information from authors about some types of materials, experimental systems and methods used in many studies. Here, indicate whether each material, system or method listed is relevant to your study. If you are not sure if a list item applies to your research, read the appropriate section before selecting a response.

Materials & experimental systems

n/a	Involved in the study
☒	☐ Antibodies
☒	☐ Eukaryotic cell lines
☒	☐ Palaeontology and archaeology
☒	☐ Animals and other organisms
☒	☐ Clinical data
☒	☐ Dual use research of concern
☒	☐ Plants

Methods

n/a	Involved in the study
☒	☐ ChIP-seq
☒	☐ Flow cytometry
☒	☐ MRI-based neuroimaging

Plants

Seed stocks	N/A
Novel plant genotypes	N/A
Authentication	N/A
